# Underwater acoustic analysis reveals unique pressure signals associated with aircraft crashes in the sea: revisiting MH370

**DOI:** 10.1038/s41598-024-60529-1

**Published:** 2024-05-02

**Authors:** Usama Kadri

**Affiliations:** School of Mathematics, Cardiff University, Cardiff, CF24 4AG UK

**Keywords:** Physical oceanography, Scientific data

## Abstract

Data analysis from the hydroacoustic stations of the Comprehensive Nuclear-Test-Ban Treaty Organization has unveiled distinctive pressure signals linked to aircraft crashes of varying sizes in the ocean. Notably, these signals were detected at distances ranging from two to five thousand kilometres, highlighting the efficacy of underwater acoustic technology in event identification and classification in marine environments. In this study, we investigate the plausibility of an aircraft, such as Malaysian Airlines Flight 370 (MH370), crashing into the sea leaving a discernible pressure signal at distant hydrophones. Consequently, we focus on recordings obtained from the hydroacoustic monitoring stations located at Cape Leeuwin and Diego Garcia, within a few minutes of the last satellite ping on the 7th arc, associated with the assumed crash time and location. Among the available data, only one relevant signal has emerged as a potential candidate, albeit recorded at a single station out of the two stations available. To ensure a comprehensive analysis, we also examine the time frame and location of the airplane along its initial route. Though no corresponding signal was observed. Nevertheless, the findings in this study narrow down the range of possibilities and present a novel scientific approach to investigate such incidents. These findings contribute to our understanding of acoustic signals associated with aircraft crashes at sea. They emphasise the potential for hydrophones to detect events even when the signal travels long distances through land. Ultimately, this research offers recommendations for conducting on-site experiments involving controlled explosions with energy levels similar to the impact of MH370 along the 7th arc. The aim is to encourage pertinent authorities to implement actions that could reveal insights into the destiny of MH370 specifically. Additionally, this initiative seeks to establish a comprehensive framework for addressing comparable incidents in the broader ocean context.

## Introduction

**Figure 1:**
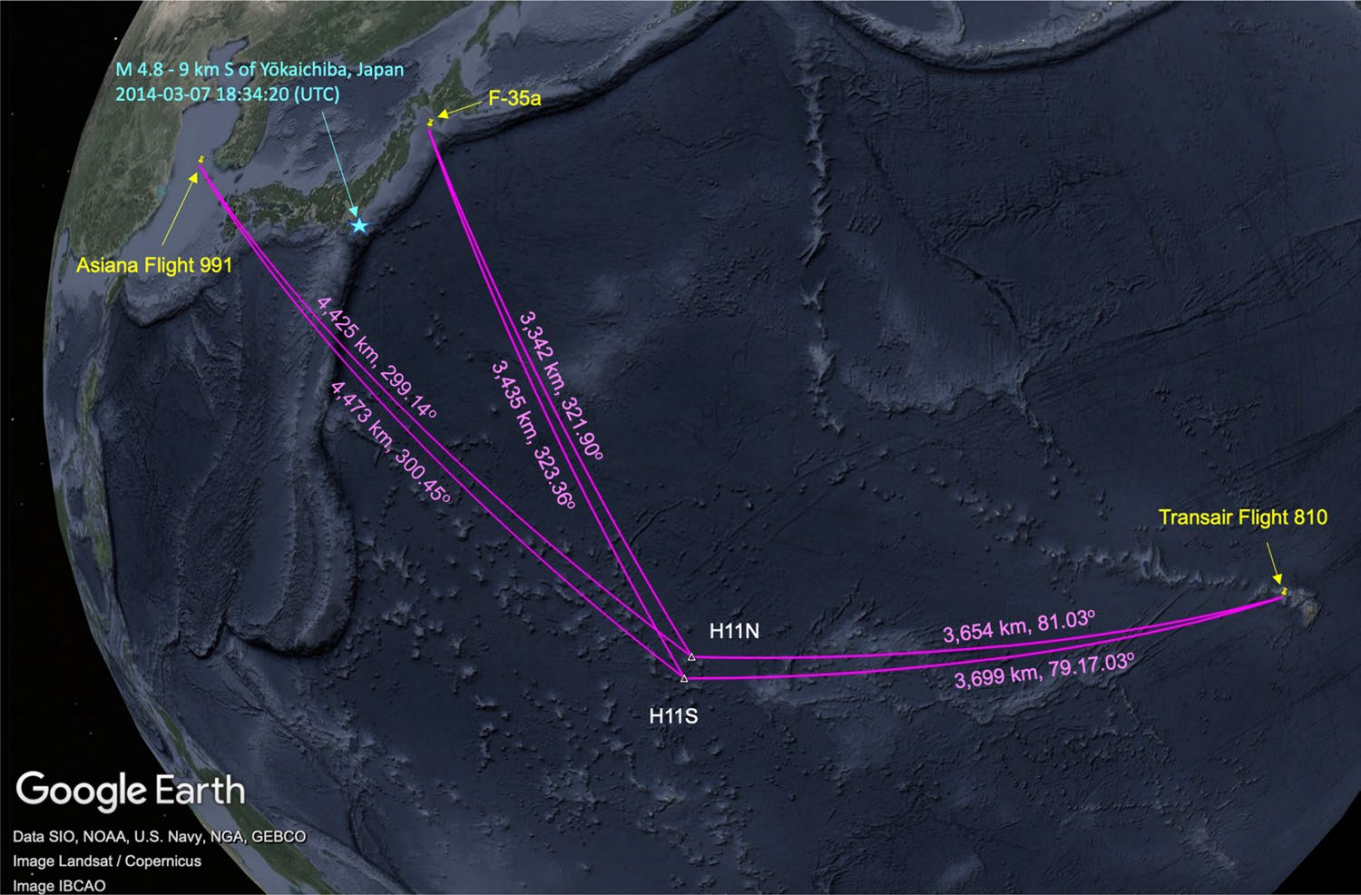
Location of the CTBTO’s hydroacoustic stations H11N and H11S (white triangles); the impact location of three aircrafts (indicated in yellow): F-35a, Transair Flight 810, and Asiana Flight 991; and the distances and bearings relative to the hydroacoustic stations (presented in magenta). The cyan star shows the location of earthquake M 4.8–9 km S of Yōkaichiba, Japan, 2014-03-07 18:34:20 (UTC) 35.611^∘^ N 140.552^∘^ E 23.9 km depth.

**Figure 2:**
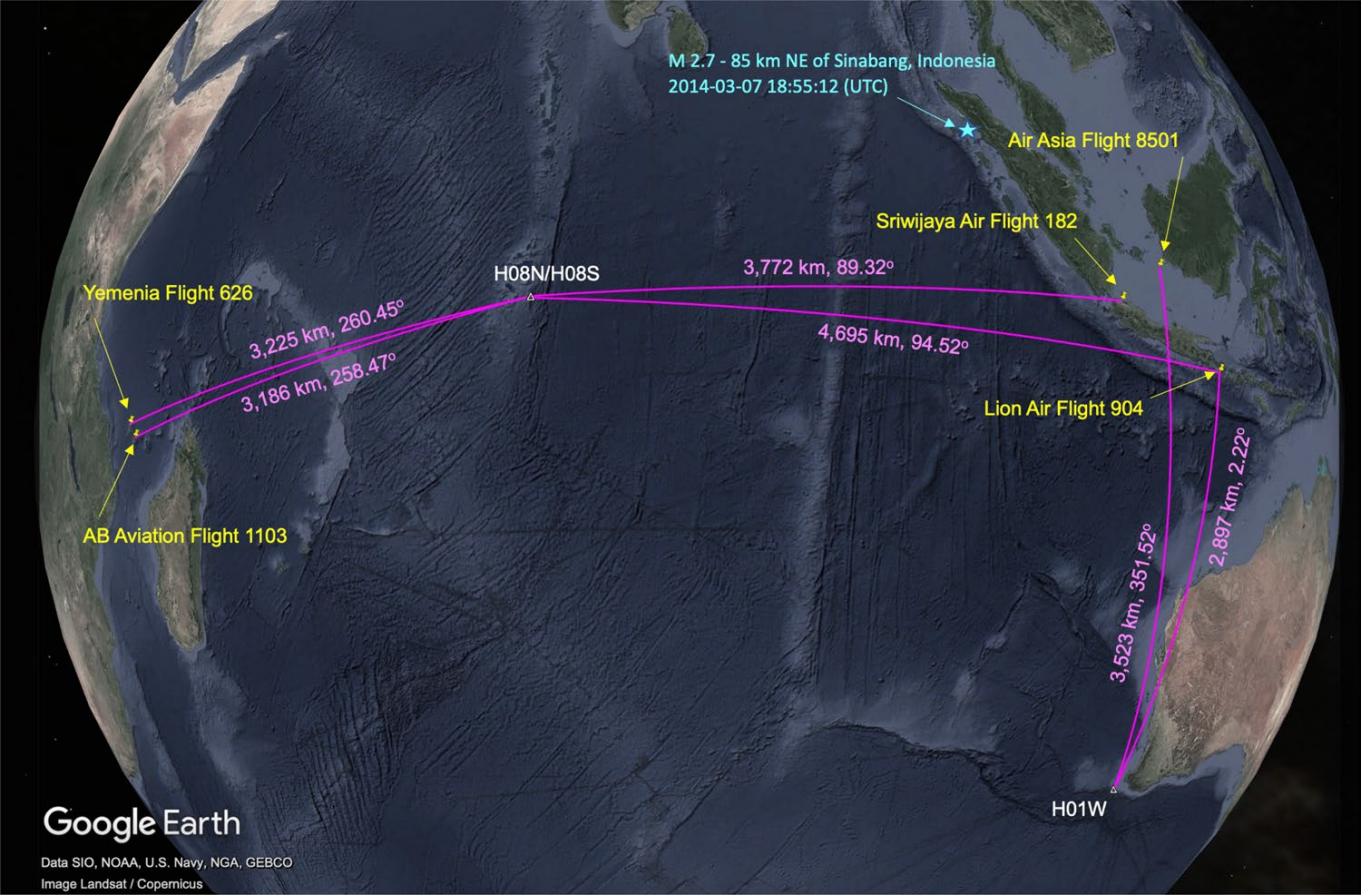
Location of the CTBTO’s hydroacoustic stations H08S, H08N and H01W (white triangles); the impact location of five aircrafts, Yemenia Flight 626, AB Aviation Flight 1103, Sriwijaya Air Flight 182, Air Asia Flight 8501, Lion Air Flight 904 (indicated in yellow); and the distances and bearing relative to the hydroacoustic stations (presented in magenta). The cyan star shows the location of earthquake M 2.7–85 km NE of Sinabang, Indonesia, 2014-03-07 18:55:12 (UTC) 3.072^∘^ N 96.873^∘^ E 10.0 km depth.

**Figure 3:**
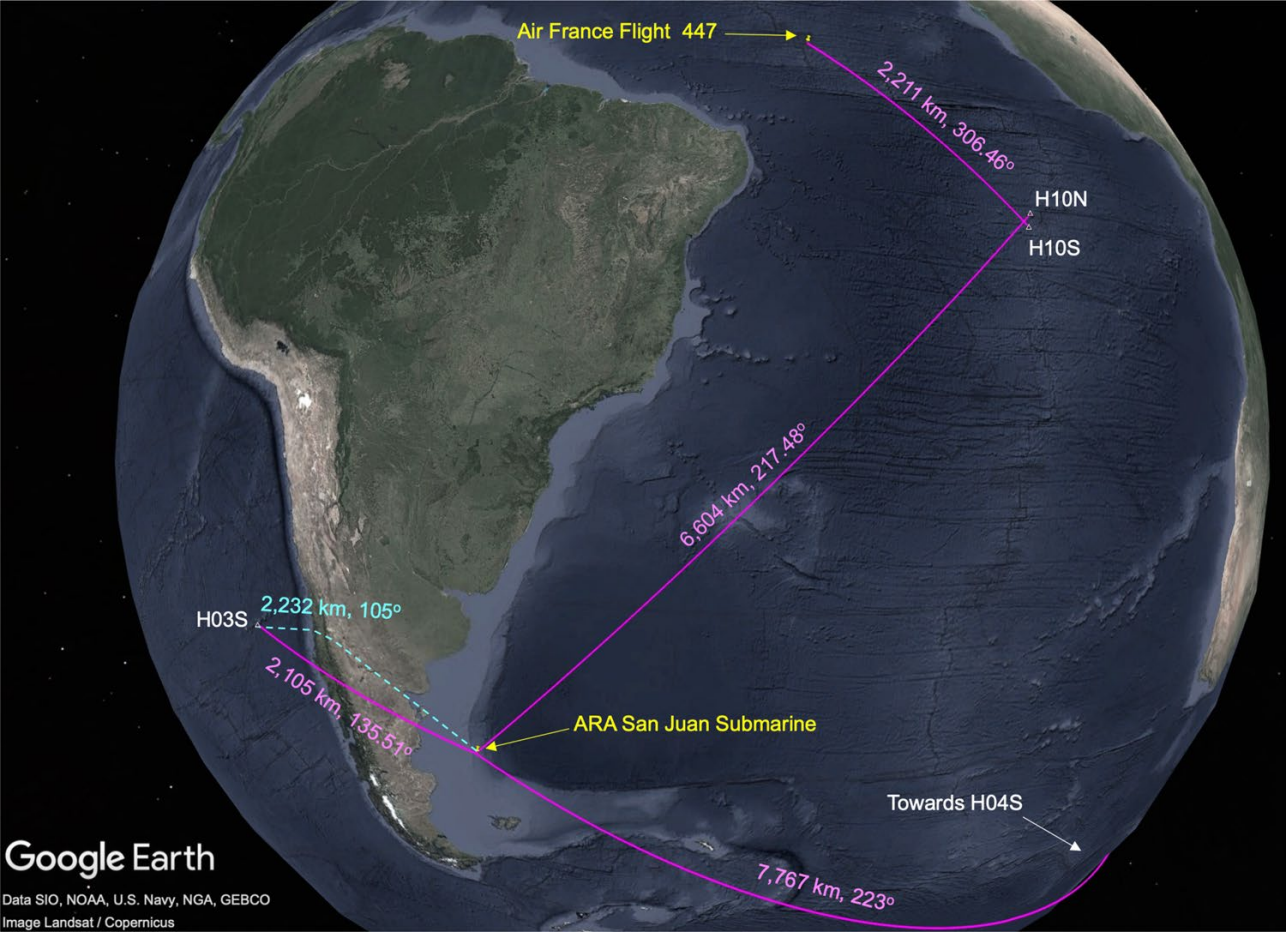
Location of the CTBTO’s hydroacoustic stations H10S, H10N and H03S (white triangles); impact location of Air France Flight 447, and disappearance location of ARA San Juan Submarine (indicated in yellow); distances and bearings relative to the hydroacoustic stations (presented in magenta); and shortest time path for the acoustic signal to travel inland (cyan).

This article delves into the perplexing enigma surrounding the Malaysian Airlines Flight 370 (MH370) by conducting an in-depth analysis of previous aircraft crashes. Out of about two decades of hydroacoustic data available from the CTBTO, a comprehensive study was undertaken of 100 hours of data identified as having the potential for containing signals pertaining to acoustic signatures of aircraft crashing into the sea. Ten historical aircraft accidents that occurred in open sea locations were selected for this study. Figures 1, 2 and 3 depict maps illustrating the positions of these aircraft and their respective distances (ranging from 2211 to 4695 km) and their bearings (directions) relative to the hydroacoustic stations. Further background information on each crash is provided in the supplementary material S1. Figure 1 showcases the impact locations of three aircraft, namely F-35a, Transair Flight 810, and Asiana Flight 991, along with their distances from the northern and southern parts of the hydroacoustic station at Wake Island, stations H11N and H11S, in the Pacific Ocean. Notably, each hydroacoustic station consists of three hydrophones arranged in a triangular configuration with distances of a few kilometres between them. By calculating the time differences in signal arrival, it is possible to determine the bearing of the signal (see “Methods”). In Fig. 2, the impact locations of five aircrafts are depicted, all of which were recorded at hydroacoustic stations in the Indian Ocean, namely Diego Garcia (H08S and H08N) and Cape Leeuwin (H01W). Yemenia Flight 626 was recorded at both H08S and H08N, while AB Aviation Flight 1103 and Sriwijaya Air Flight 182 were recorded at H08S. Air Asia Flight 8501 was recorded at H01W, and Lion Air Flight 904 was recorded on both H01W and H08S, providing a rare opportunity to study signal variations due to propagation through land. Low-frequency acoustic signals coupling to land and subsequently to the ocean have been reasonably well-documented^1,2^. The concept that hydroacoustic or more generally acoustic-gravity wave signals originating from objects impacting the sea surface can transition between land and water has been proposed by Ref.^3^. To demonstrate the detectability of such signals even after traveling hundreds of kilometres inland, the case of the ARA San Juan, an Argentine submarine that vanished off the coast of Argentina on November 15, 2017, is considered. An explosion at the disappearance site was detected on CTBTO stations Ascension (H10) and Crozet Islands (H04), located thousands of kilometres away^4^. In Fig. 3, the location of the submarine and the vast stretch of land separating it from the Juan Fernandez Island hydroacoustic station (H03S) is depicted. A similar distance, but without any land in between, separates Air France Flight 447 from H10S.

## Past aircraft crashes with known locations

**Figure 4:**
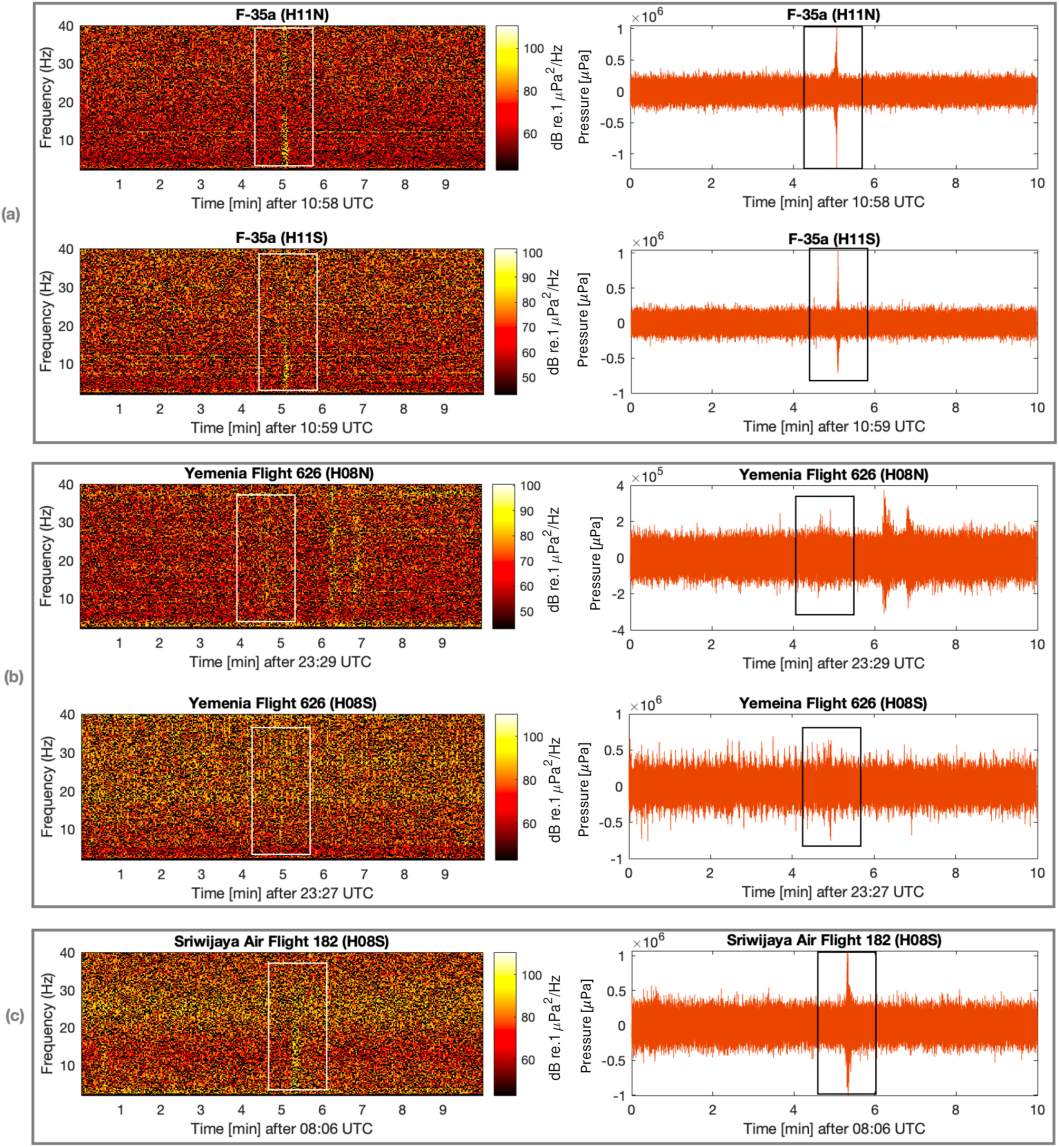
Spectrogram (left) and pressure time series (right) of: (**a**) F-35a; (**b**) Yemenia Flight 626; and (**c**) Sriwijaya Air Flight 182. Rectangles highlight the signals of interest.

**Figure 5:**
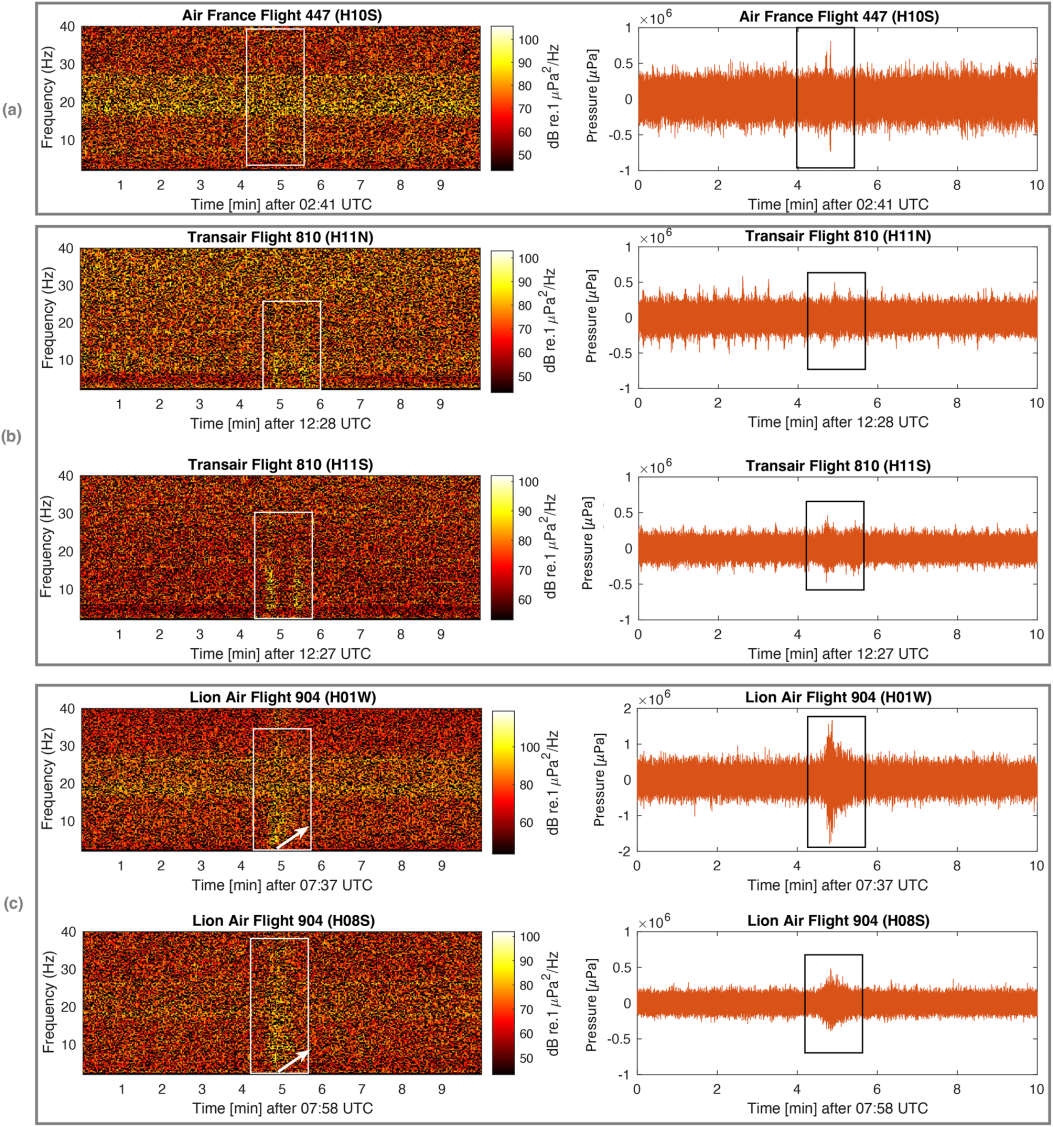
Spectrogram (left) and pressure time series (right) of: (**a**) Air France Flight 447; (**b**) Transair Flight 810; and (c) Lion Air Flight 904. Rectangles highlight the signals of interest, and white arrows highlight dispersion.

**Figure 6:**
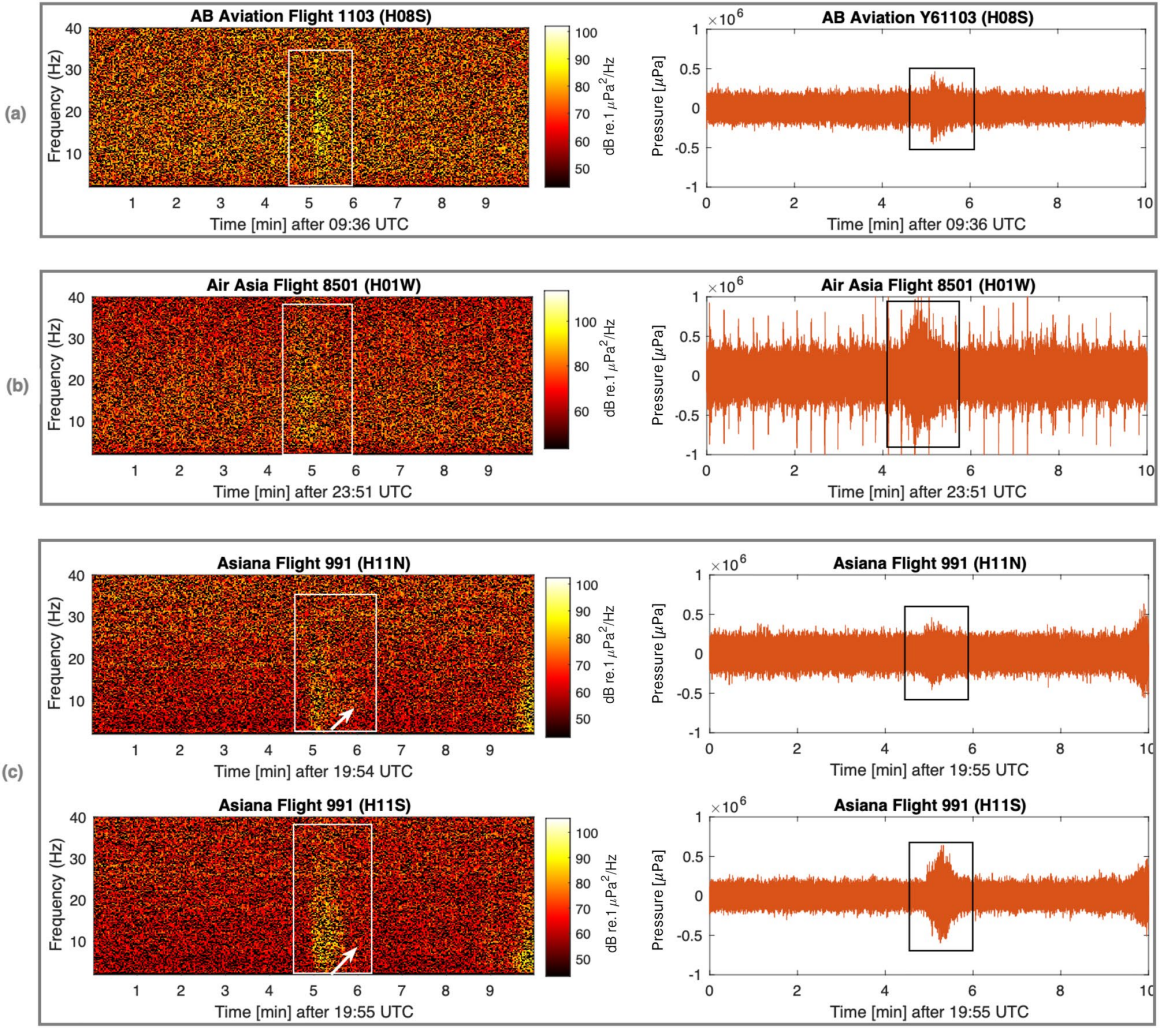
Spectrogram (left) and pressure time series (right) of: (**a**) AB Aviation Flight 1103; (**b**) Air Asia Flight 8501; and (**c**) Asiana Flight 991. Rectangles highlight the signals of interest, and white arrows highlight dispersion.

The spectrograms and pressure time series of signals from ten aircraft incidents and a single submarine disappearance, recorded on seven hydroacoustic stations of the CTBTO, are presented in Figs. 4, 5 and 6. Detailed information about each incident can be found in the supplementary material S1, and technical notes on the calculation methods employed are given in the “Methods” section. A consistent observation in this study, across all aircraft incidents, is the detection of acoustic signals even at distances of thousands of kilometres from the hydrophones.

The spectrograms of aircraft F-35a exhibit a distinctive, sharp, and rapid (6 s) signal following the supersonic impact (Fig. 4a). This signal is captured at both stations, H11N and H11S, which are over 3300 km away. The result is in agreement with Ref.^5^ who studied this event in detail. A similar pattern is observed in the case of Yemenia Flight 626, also recorded at two stations, H08N and H08S (Fig. 4b), although the signal appears fainter compared to F-35a, likely due to a less energetic impact. It is worth noting that the background noise at H08S is particularly high, posing a challenge for isolating the signal of interest. Conversely, H08N displays two subsequent signals that appear to be local events. For Sriwijaya Air Flight 182 (Fig. 4c), data was only available at H08S within the specified time window. Despite the significant distance from the hydrophone station, the signal of interest is sharp and clear.

The signal associated with Air France Flight 447 (Fig. 5a) is also short, but due to relatively high background noise between 18 and 28 Hz, the signal appears faint. It is important to emphasise that Flight 447 experienced a high-impact event, as evidenced by the pressure values reaching 75% of that observed for F-35a, even over a distance of 2200 km. Note that the above mentioned flights had powerful impacts, some due to aerodynamic stall prior to crashing.

The incident involving Transair Flight 810 is noteworthy as data is available from both H11N and H11S (Fig. 5b), and there appears to be a double signal, potentially indicating a ditching event, an engine that was detached, or signal arrival from different locations. The duration of the signal is longer compared to previous cases, suggesting either signal dispersion (When sound propagates through the ocean, it encounters variations in water temperature, pressure, and salinity. These environmental factors can lead to changes in the speed of sound at different depths and locations. As a result, different frequency components of a sound signal may travel at different speeds, causing the signal to spread out or disperse over time. This phenomenon is known as dispersion.) or an extended impact time due to ditching. A similar observation, with more pronounced dispersion indicated by white arrows, is noticed in the case of Lion Air Flight 904 (Fig. 5c). The associated signals, within the expected time windows, were recorded at two distant stations, namely H01W and H08S. Notably, station H08S is nearly twice the distance from the incident location, resulting in stronger signal dispersion. The shape and arrival time of the signal at H01W indicate that it primarily travelled through water, although the bearing suggests a landward direction. The high pressure recorded at H01W indicates that the signal might be associated with a different event, possibly local, especially considering reports of Lion Air Flight 904’s relatively low-impact crash. It is also noteworthy that approximately 8 min later, two faint signals (not shown in the figure) were observed from feasible bearings, indicating travelling predominantly in the water layer.

In the final set of plots, Fig. 6, signal dispersion is most pronounced. Panels (a), (b), and (c) of Fig. 6 present signal recordings associated with aircraft incidents involving AB Aviation Flight 1103 (recorded at H08S), Air Asia Flight 8501 (recorded at H01W), and Asiana Flight 991 (recorded at both H11N and H11S). Nevertheless, the differences in the mode of crash is rather pronounced. The signal in Fig. 6b is distorted with noise (possibly due to airgun shots as shown in Figs. 3(a) and 4(c) of Ref.^6^), and the magnitude of the pressure induced by the aircraft crash is not clear. AB 1103 is a small aeroplane, whereas Asiana 991 (a heavy cargo aeroplane) and Air Asia 8501 had a much more impactful crash.

In summary, the analysis of the recorded signals from past aircraft incidents reveals three different types of acoustic signature. The first type is characterised by distinct and rapid signals following energetic impacts, whereas the second type includes signals with longer impact time. The third type involves signals with higher uncertainty around them, due to multiple factors such as large distances crossing through land and unknown modes of impact (e.g., nose, main body, or wings first). However, the pressure signatures radiating during a high energy impact crash were found to be observable even at a few thousands kilometres away. The effect of signal travelling through land is discussed in the following note considering the case of ARA San Juan Submarine.

**Figure 7:**
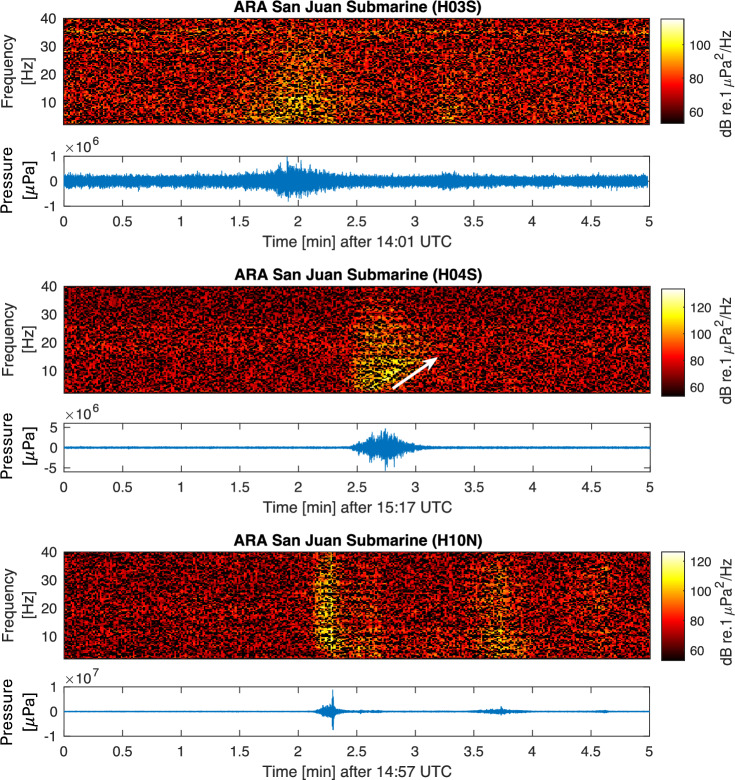
Spectrogram and pressure time series of ARA San Juan Submarine explosion recorded at stations H03S (top), H04S (middle), and H10N (bottom). White arrow highlights dispersion.

**Figure 8:**
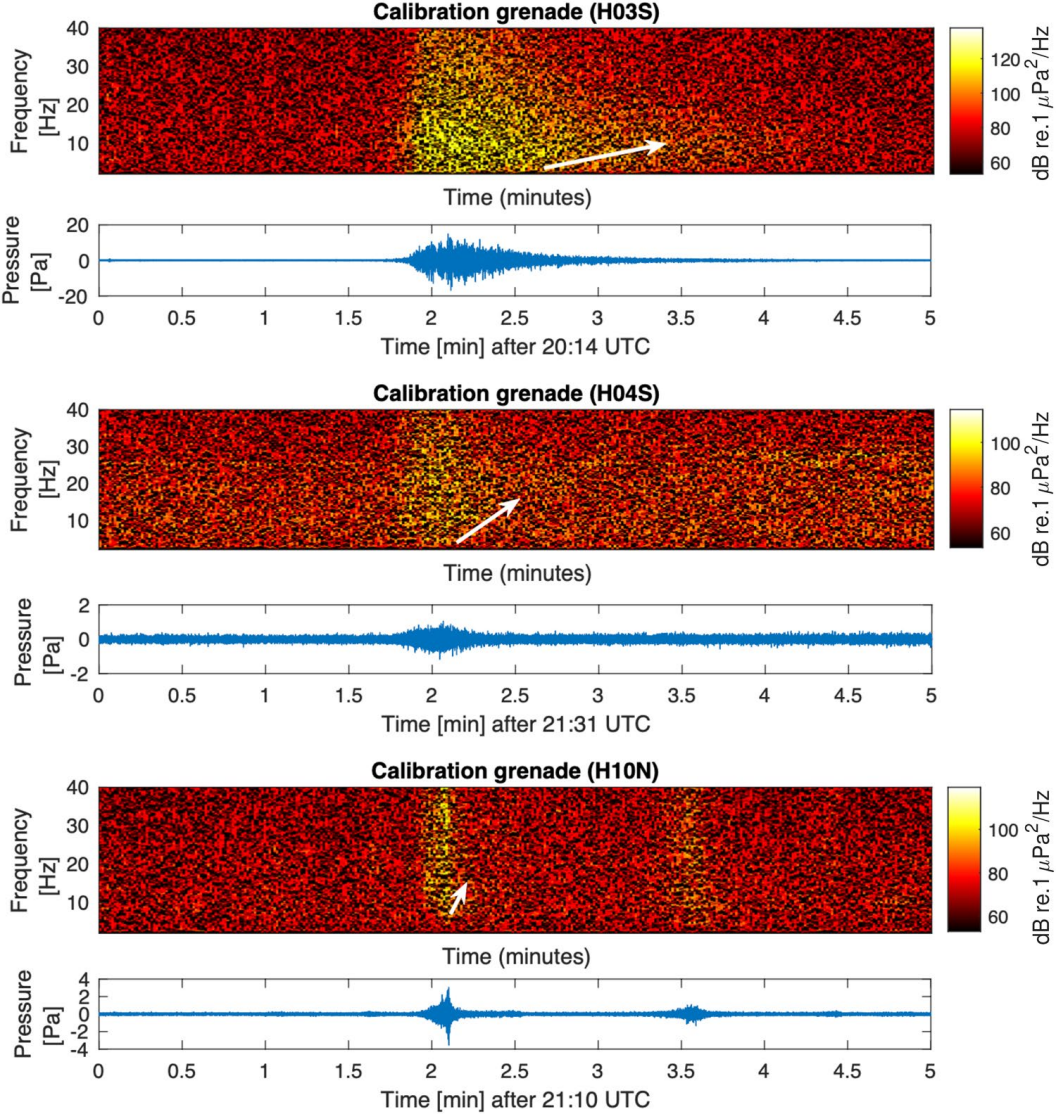
Spectrogram and pressure time series of a calibration grenade recorded at stations H03S (top), H04S (middle), and H10N (bottom). White arrow highlights dispersion.

### A note on ARA San Juan Submarine

The ARA San Juan Submarine case is a good example for demonstrating that acoustic signals that radiate in water can couple to the elastic seabed when approaching shallow water, then travel large distances in land before returning to the sea where they can still be recorded at hydrophones. Specifically, Fig. 7 presents spectrogram and pressure time series of acoustic waves generated by explosions in the submarine that were observed not only at H10 and H04 stations that have direct water connection with the location of the submarine (which is in agreement with Fig. 2 of Ref.^4^), but also at station H03, that is separated by 2000 km of land as illustrated in Fig. 3. This observation highlights the effectiveness of employing hydrophones to capture events in the ocean beyond current convention, namely when signals cross through lands. However, the higher frequencies of the signal attenuate due to scattering absorption in the elastic layer when a critical depth is reached. This observation is in agreement with acoustic-gravity wave theory^7,8^: while higher acoustic modes might be absorbed in the elastic layer when a critical water height is reached, the leading mode couples with the elastic sea-bed, turns into Scholte wave, then Rayleigh-type wave, and thus preserves most of the energy. It is noticed even though H03S is much closer to the event location compared to the other two stations, i.e., about one third the distance to H10N and one quarter the distance to H04S, yet the pressure signal recorded at H03S is 21% of H04S and 11% of H10N. Thus, radiation through large distances in land and complex topography can significantly reduce the pressure signature.

Moreover, the shorter propagation path in terms of duration, not necessarily the shortest distance, experiences less attenuation during transmission, resulting in a greater concentration of acoustic energy. The cyan curve in Fig. 3 qualitatively indicates the path the acoustic signals would travel through land, which minimises the duration of the journey as it optimises travelling in the solid earth (much higher speed) than in the water. Specifically, with an average speed of 3550 m/s travelling roughly 1900 km $\pm 100$ km, and an average speed of 1480 m/s travelling about 330 km $\pm 100$ km, it takes a total time of between 12 and 13.5 min to reach H03S. This choice of shortest time path results in a different relative bearing, i.e., 105.6^∘^, which is in agreement with the actual arrival direction at H03S (with an uncertainty of 0.5^∘^), as opposed to the expected 135.5^∘^, in case of shortest distance or 173^∘^ considering the acoustic signals travelling only in the water.

Further support of the argument above can be demonstrated by examining an event that took place in close proximity to the site of the submarine’s explosion. On December 1, 2017, 2 weeks after the ARA Juan submarine vanished, the Argentine Navy dropped a calibration grenade into the sea within the vicinity of the submarine’s last known location. Comparing the temporal and spectral features of both events helps confirming the impulsive nature of the San Juan event, as rigorously done by Ref.^4^. Here, the grenade event provides a further validation (Fig. 8) that acoustic signals in water can travel across large distances in land before attenuating significantly, in particular for lower frequency modes^3,8^. Figure 8 presents spectrogram and pressure time series of acoustic waves generated by the grenade. As before, the signals observed at H10 and H04 stations are in agreement with Fig. 2 of Ref.^4^, and the signal recorded on station H03 matches the expected arrival time of 13 min after the explosion, the duration being 30 s, and the frequency range and the secondary smaller signal that appears exactly 1.5 min after the main signal, just as observed in Fig. 7 for the ARA San Juan Submarine case.

## The unsolved case of MH370

The Malaysia Airlines Flight 370, known as MH370, captivated the world upon its disappearance on March 8, 2014, during a journey from Kuala Lumpur to Beijing. Despite an extensive multinational search effort, the whereabouts of the aircraft and the fate of its 239 passengers and crew remain shrouded in uncertainty. To delve into the perplexing enigma surrounding MH370, attention is focused on Southern Indian Ocean, which is, arguably, associated with the final stage of the journey, searching for signals that were generated near the 7th arc (last communication with the satellite - Inmarsat) following the official search recommendations. In addition, data associated with the disappearance stage of the flight around the time of the last communication, i.e., in the Gulf of Thailand, was analysed in general to check for any unusual signals. No signals of interest were found to be associated with the early disappearance stage (see supplementary material S1).

### The final search stage: southern Indian Ocean

According to the official search team, data that emerged later on from the International Maritime Satellite Organization (Inmarsat) alongside calculations by Boeing search teams concluded that the MH370 crash impact has to be in the Indian Ocean at the vicinity of the last handshake at 00:19:29 UTC between Inmarsat and MH370, known as the 7th arc^9^. Moreover, it has been concluded that during the last minutes of the flight the aircraft was on autopilot at regular altitude of 36,000 ft when the two engines had subsequently flamed off, following fuel being exhausted, which caused the aircraft go through a spiral stall. Consequently, the crash impact was highly energetic, as also supported by evidence from debris^9^. Bayesian methods employed for analysing potential routs support these findings as well^10^. Provided all these information not only that the time window and bearing being sought are very narrow, but one expects a significant signal to appear on both H01W and H08S (note that H04 was not operational during that time, and until 2017). The distance from the 7th arc to H01W is about 1600 km, which is less than three quarters the distance of Air France Flight 447 relative to H10S (the signal-to-hydrophone distance analysed); and the distance to H08S is around 3700 km, still within the average distance of the other studied cases.

**Figure 9:**
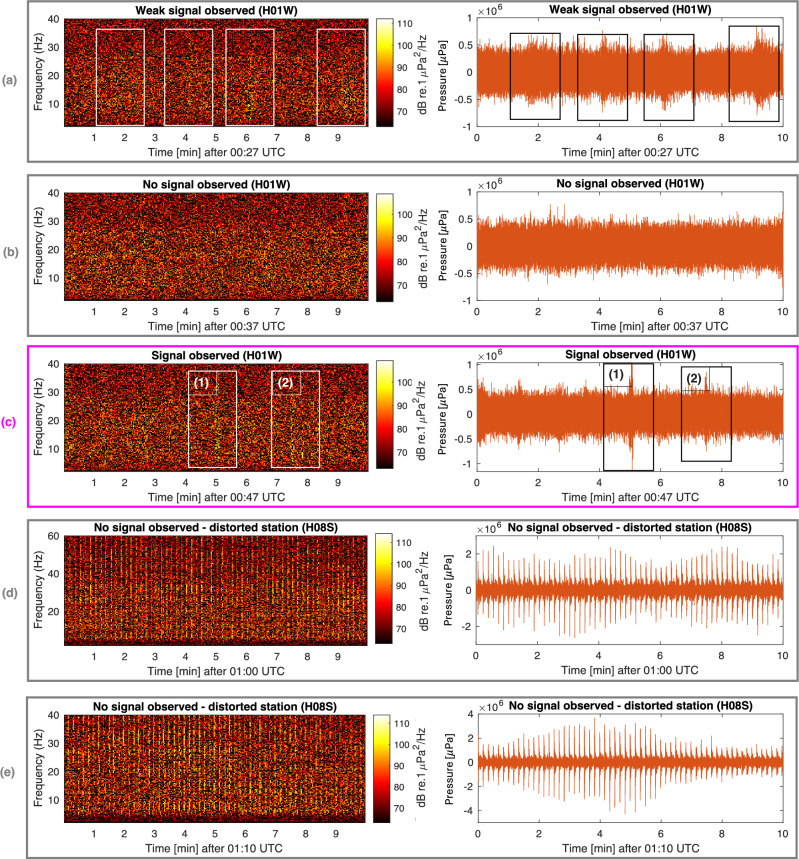
Spectrogram and pressure time series of signals observed at H08S and H01W on 8 March 2014 shortly after the last handshake (7th arc) of MH370. The only signal of interest is found in rectangle (2) of panel (**c**), which has a bearing of 306^∘^ relative to H01W; signal in rectangle (1) has a bearing of 57^∘^. For a comprehensive list of signals shortly after the 7th arc see Table 1.

A number of acoustic signals were analysed previously in several studies, in particular Refs.^11,12^. One of these, a signal of interest with a bearing of 301.4$\pm 0.4^{\circ }$ relative to H01W, was recorded at 01:34:40 UTC on 8 March 2014. A later study by Refs.^3,13^, suggested that the signal is at distance of 1900 km ($\pm 200$ km) from H01W, centred at $-23.662^{\circ }$, $96.676^{\circ }$, with the source generated between 01:11 and 01:16 UTC on 8 March 2014). However, following the recommendations by the official investigation the signal of interest that is associated with the impact has to occur around the 7th arc, both in time and location. Around 00:30 UTC there are a few repeating signals (Fig. 9a) followed by no observed signals (Fig. 9b), around the requested time window. After that, there are only two signals that were observed within the requested time window, as shown in Fig. 9c. The first signal has a bearing of $57^{\circ }$ and thus cannot be related to MH370, but the second, which was recorded at 00:52 UTC at H01W, arrives from a plausible direction, i.e., $306^{\circ }$. Notably, the transect from the 7th arc to H01W has no bathymetric irregularities (see Fig. 15c in “Methods”), and thus minimum loss of energy is expected due to scattering. Confirming the same signal of interest at H08S would be a challenging task if at all possible due to the very high noise distortion (most likely airgun), and the significant bathymetric rise midway along the transect, that is expected to significantly reduce the transmission of the signal. The expected pressure amplitude at H08S, if had a similar value as recorded on H01W, would be 0.5 [Pa], whereas the airgun noise is ranging from $2-4$ [Pa] which is 4–8 times stronger (Fig. 9d–e).

In summary, adhering to the official narrative, it can be asserted with confidence that if MH370 did crash in the vicinity of the 7th arc, then hydroacoustic signals should have been detected at least at H01W. Within the specified time window, only a single signal of interest at a bearing of $306^{\circ }$ has been identified.

**Figure 10:**
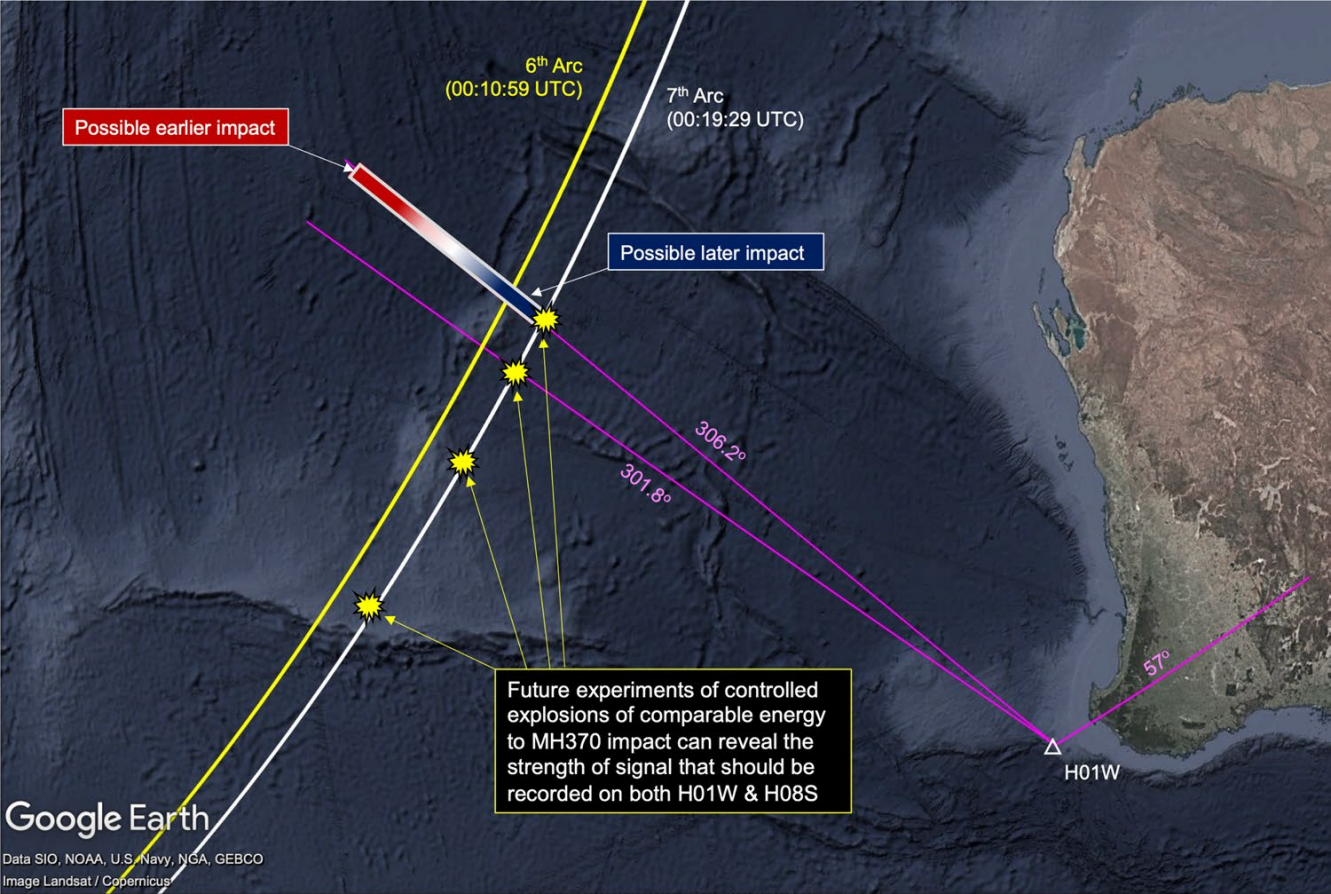
Location of the CTBTO’s hydroacoustic station H01W (white triangle); Inmarsat 6th arc at 00:10:59 UTC (yellow curve); Inmarsat 7th arc at 00:19:29 UTC (white curve). The possible impact location of the source of signal (2) of Fig. 9c is presented in a gradient rectangle (red for earlier impact, and blue for later impact). Suggested future controlled explosions of comparable energy to MH370 are illustrated by yellow explosion symbol.

## Discussion and practical suggestions

The limited number of analysed signals from aircraft crashes in this study hinders a comprehensive examination of acoustic signatures arising from various modes of aircraft crashes occurring under complex conditions, including diverse topography and the presence of natural and manmade noise sources. Nonetheless, it is implausible to imagine that a significant crash of an aircraft on the ocean surface would fail to generate a discernible pressure signature, even at distant hydrophones, let alone those in closer proximity. The official investigation of MH370 supports the conclusion that a substantial crash should have taken place near the 7th arc^9^. In this case, the acoustic signal would have travelled approximately 1600 km to reach the nearest hydrophone station (H01W), a considerably shorter distance than any other acoustic signal analysed here. An aircraft with a mass of 200 tons impacting the water surface at a velocity of 200 m/s possesses a kinetic energy of 4 GJ, equivalent to 956 kg of TNT or an earthquake of magnitude 3.2. Considering half that velocity, the released energy would still be large, i.e., 1 GJ, equivalent to 239 kg of TNT or an earthquake of magnitude 2.8. Even with a significantly lower impact velocity of 30 m/s, the resulting energy release would be 89 MJ, equivalent to 21 kg of TNT or an earthquake of magnitude 2.1. Evidently, such earthquakes can be detected by distant hydrophones, just as the M 2.7 earthquake (see Fig. 2 for the location of the earthquake on the map; and Fig. 17 in “Methods” for the bearing). Therefore, it is highly unlikely for MH370 to have crashed near the 7th arc without leaving a discernible acoustic signature. Within the constraints imposed by the official search team, only one signal (bearing $306^{\circ }$) has been identified at H01W, suggesting a potential impact location (see “Methods”), as illustrated in Fig. 10. However, this same signal was not observed at H08S. Whether the signals at bearings $306^{\circ }$ and the later signal at $301^{\circ }$, are related remains subject to future analysis.

A practical suggestion to help resolve the ongoing debate regarding the detectability of the acoustic signal radiated by MH370 is to conduct controlled explosions along the 7th arc (similarly to ARA San Juan Submarine^4,14^), containing an equivalent amount of energy believed to be associated with MH370. If the received signals from the explosions exhibit comparable pressure amplitudes to that of the signal of interest, that would support the notion that the identified signal should be a focal point in future search efforts. Conversely, if the signals received on both H01W and H08S are significantly stronger than the signal of interest discussed here, it would necessitate further analysis of the signals from both stations. This may also warrant a reassessment of the data that led to the determination of the 7th arc, allowing for the consideration of new scenarios consistent with the updated findings. Additionally, variations in the strength of recorded signals could offer insights into the underlying conditions influencing such variability, potentially enabling a more refined localisation of potential impact areas based on specific terrains and associated transects.

It is important to note that the present study does not definitively pinpoint the impact location of MH370. However, it significantly enhances our understanding of the acoustic signals associated with aircraft crashes at sea in general. It narrows down the signals of interest and their respective locations, and importantly, it highlights the effectiveness of hydrophones in classifying and detecting signals even after they have travelled long distances, including over land. It is hoped that these practical suggestions will inspire relevant authorities to take the next significant steps towards unraveling the greatest aviation mystery of all time.

## Methods

### Analysis approach

Conducting a detailed analysis of aeroplane crashes in the ocean is challenging due to the limited number of occurrences. Despite the scarcity of data, the unique and energetic nature of such events, coupled with a relatively low frequency of occurrence of signals with similar characteristics, presents an opportunity for insightful analysis. For instance, the highest relevant signal frequency observed in the analysis was four signals in 10 min, in Fig. 9a, highlighting a constrained set of possibilities. Considering the 10-min window and allowing for a 2^∘^ uncertainty in the bearing (which is much larger than the maximum deviation), the probability of a signal occurring by chance, unrelated to the event, is less than 3%. This high level of confidence (97%) in the signal’s relevance to the event arises from the limited occurrences and distinctive characteristics of the signals. Even with a broader 20-min window, featuring eight signals and a more lenient 5^∘^ bearing uncertainty, the probability of the signal being unrelated to the event remains below 12%. However, it is acknowledged that the disadvantage of this approach emerges when the impact location is unknown, leading to an increased degree in bearing uncertainty.

It is worth noting that in the calculation of the acoustic speed, although average speeds can be calculated with precision, the methodology employed here takes a broader perspective. Rather than focusing solely on accuracy, the approach involves exploring the entire spectrum of possibilities, even if it means incorporating seemingly irrelevant signals. This ensures that no potential signal of interest is inadvertently overlooked. Therefore, prior to diving into the sufficient conditions to correlate the signal with the incident, the necessary conditions are first examined. This approach is chosen due to the limited number of signals that meet the necessary conditions as described above.

### Signal processing and bearing calculation

Signals from past aircraft crashes were presented around the middle of a 10 min time window, to allow visual comparison. Spectrograms indicate the frequency band of noise as opposed to frequency bands from signals. Most processed signals show a significant amount of noise in the 0–4 Hz band. Noting that the broadband frequency content is typical for the propagation of acoustic-gravity waves^15^ that can travel far distances from the event location, a high pass Butterworth IIR filter was used (in addition to the CTBTO high pass filter) below 5 Hz. Moreover, a 2–40 [Hz] band-pass filter was applied in general. Filtering noise, enhancing the signal appearance and stabilising the time-waveform can be crucial for identifying the bearing of the signal of interest, in particular when the source is an impulse that generates short signals of a few seconds length.

Each hydrophone station has three hydrophones configured in a triangular shape spaced by around 2 km. Exact locations of all hydrophones can be obtained from the CTBTO. In information theory, entropy serves as a measure quantifying the amount of information encapsulated within a signal. Let $P(x)$ denote the probability distribution function of the discrete-time signal $x$, then the entropy is defined as

1 $$\begin{aligned} H(x) = - \sum P(x) \cdot \log _2 P(x). \end{aligned}$$

Additionally, log energy entropy^16^, represented as $H_L$, is defined as:

2 $$\begin{aligned} H_L = - \sum P(x) \cdot \log _2 P(x). \end{aligned}$$

**Figure 11:**
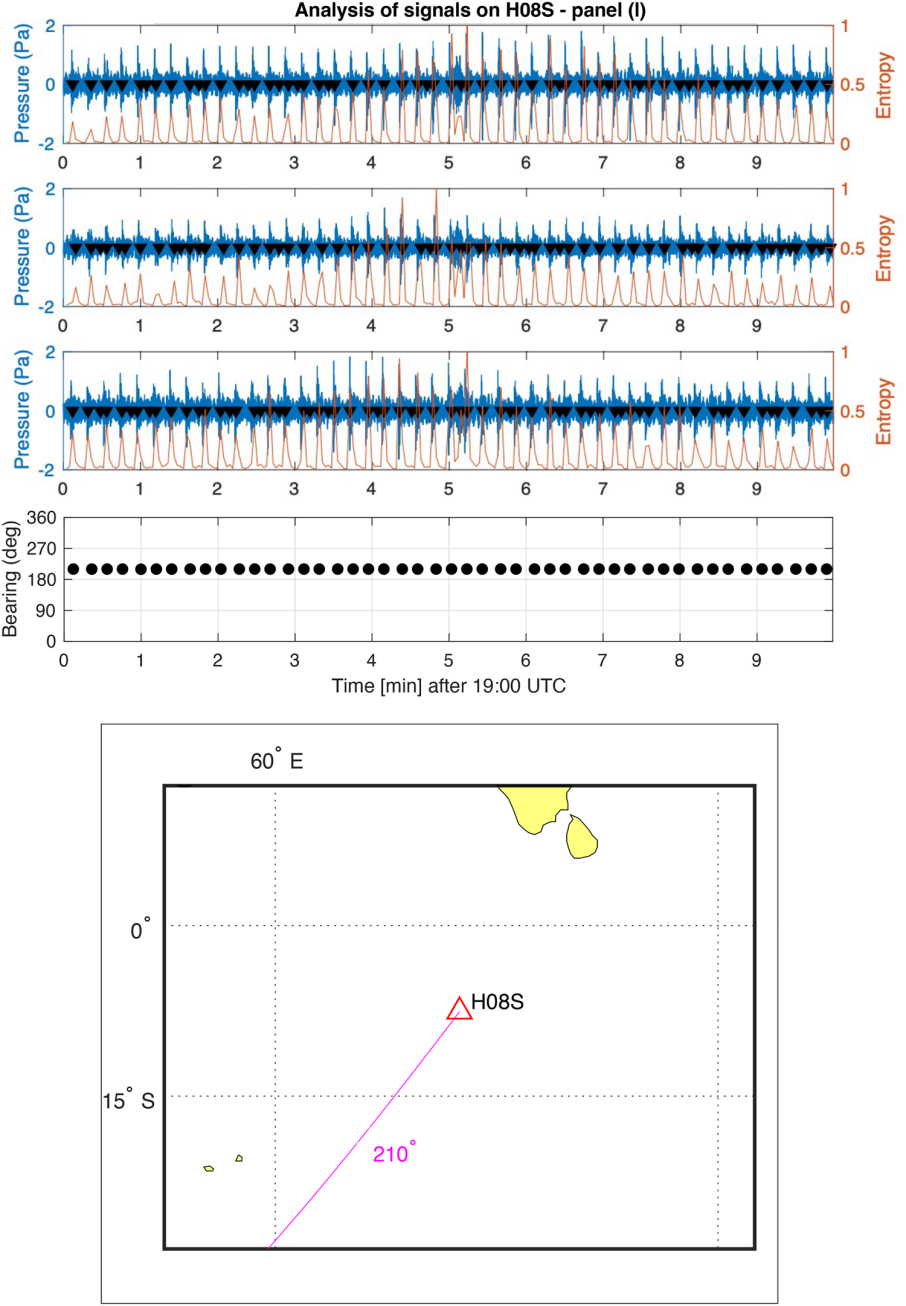
Bearing calculations of signals on Fig. S1c. From top to bottom: recordings from three channels at station H08S between 19:00 and 19:10 UTC on March 7th 2014. Windowed entropy shows peaks (black triangles) where a transient signal is found. Each peak defines an event, for which bearing is calculated from differences in arrival times in the bearing subplot. The map demonstrates the dominating bearing from airgun signals, $210^{\circ }$ (highlighted in magenta), which prevent calculating the bearing of the signal of interest at 19:05 UTC.

**Figure 12:**
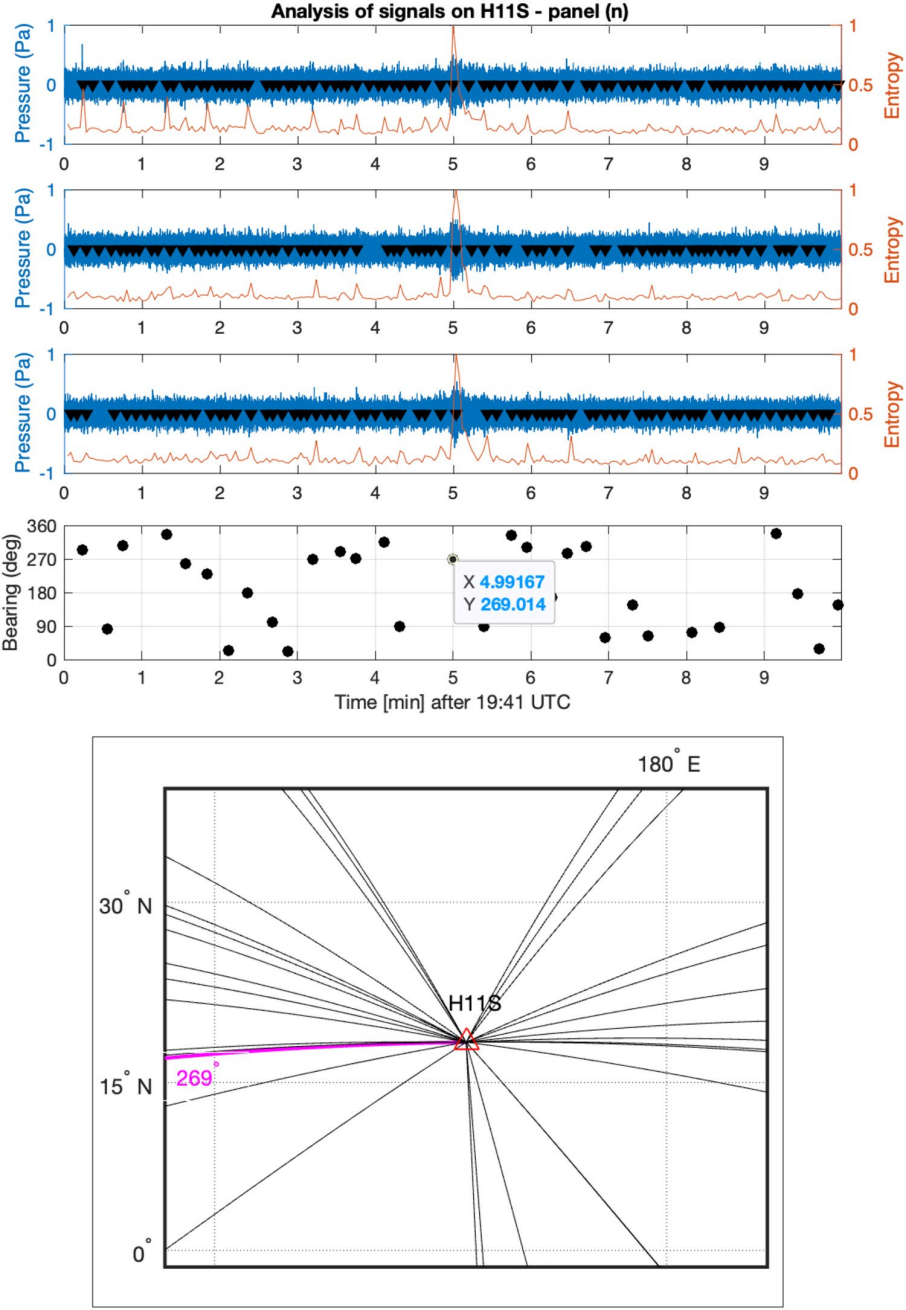
Bearing calculations of signals on Fig. S1e. From top to bottom: recordings from three channels at station H11S between 19:41 and 19:51 UTC on March 7th 2014. Windowed entropy shows peaks (black triangles) where a transient signal is found. Each peak defines an event, for which bearing is calculated from differences in arrival times in the bearing subplot. The map illustrates the direction of the calculated bearings. The bearing of the signal of interest at 19:46 UTC is successfully calculated as $269^{\circ }$ (highlighted in magenta).

Windowed entropy calculations can be employed to identify transient signals against a noisy background. This is predicated on the assumption that the randomness measure of the signal will exhibit changes when the nature of the signal undergoes alterations. Entropy values have been computed across a window size of a few seconds and a step size of 0.5 s. As depicted in Figs. 11 and 12, peaks in the entropy trace emerge where transient signals are detected. A threshold of $2.3 \times 10^4$ has been established; all peaks within a 2-second window across all signals are considered for subsequent bearing calculations. Note that the frequencies of the signal of interest, are within a frequency band, say below 20 Hz, which is order of magnitude lower than the sampling rate of the hydrophones (250 Hz). Thus related errors are expected to be small^17^.

**Figure 13:**
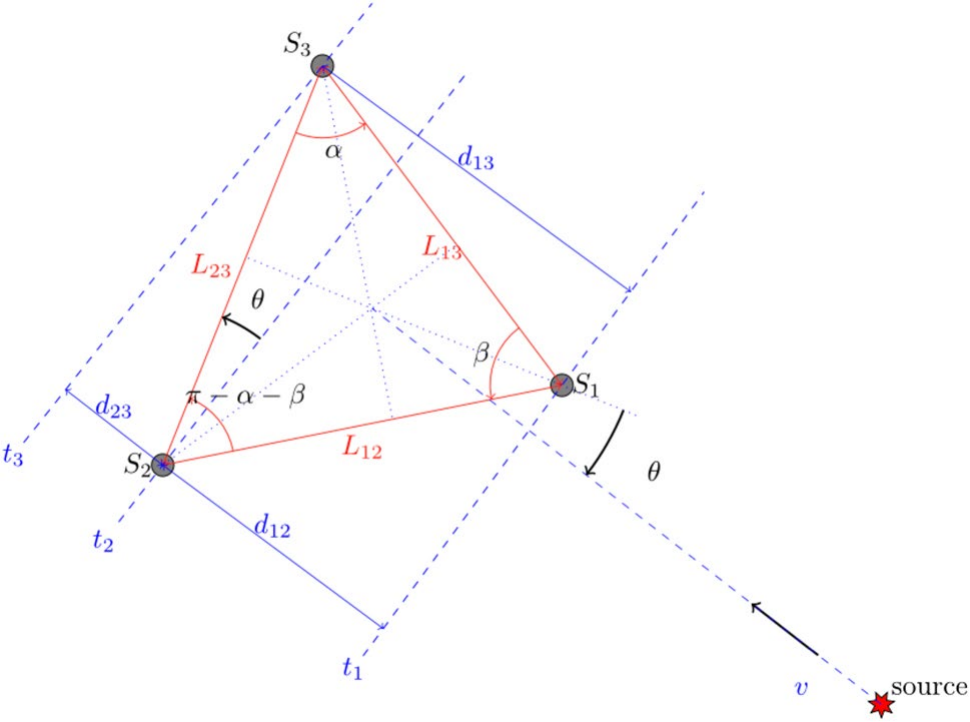
Geometry of the three hydrophones array and bearing calculation. Credit: Fig. 9 of Ref.^13^.

Following the isolation of signals of interest, bearing determination is carried out using time-of-arrival-based triangulation. The time of arrival is estimated by identifying the maximum of the cross-correlation function across channel pairs, enabling the derivation of pairwise time of arrival differences $t_i - t_j = \Delta _{ij}$. Geometric parameters for the array are derived from latitude and longitude position data corresponding to the hydrophones. Assuming a constant arrival velocity *v* an expression of the geometric parameters is given by^13^

3 $$\begin{aligned} \theta = \cot ^{-1}\left[ \frac{L_{23}}{L_{12}\sin (\alpha +\beta )}\left( \frac{\Delta _{21}}{\Delta _{32}}-\frac{L_{12}}{L_{23}}\cos (\alpha +\beta )\right) \right] \end{aligned}$$

with parameters defined in Fig. 13, with no loss of generality. Using a 99.5% confidence interval, the bearing calculations are considered to be accurate to $\pm 0.4^{\circ }$ (see Ref.^13^ for more details).

Figure 11 illustrates how high noise to signal ratio can prevent calculating the bearing of the signal of interest. In particular, the figure concerns bearing calculations of signals from airgun shots on Fig. S1c. Clearly, the airgun bearing of $210^{\circ }$ (highlighted in magenta) dominates the picture, which prevents calculating the bearing of the signal of interest at 19:05 UTC. On the other hand, Fig. 12 provides a successful capture of the bearing of the signal of interest $269^{\circ }$ (highlighted in magenta) 19:41 UTC.

### Possible impact location near the 7th Arc

A comprehensive list of signals potentially recorded near the 7th arc shortly after the last handshake are given in Table 1. The only major signal has a bearing of $306.18^{\circ }$, and it is recorded at 00:54 UTC. In that direction the distance of the 7th arc from H01W is $R_7=1586$ km. For an acoustic signal travelling with a speed of $c=1500 \,{\pm \,70}$ m/s, the required time to travel from the 7th arc to the H01W is $17.45\pm 0.65$ min. The time difference between the 7th arc and the time the signal was recorded is 34.5 min. Thus, if the signal is associated with the aircraft crash then there is an extra time of $\Delta t = 17.02\pm 0.65$ min. Therefore, if the airplane was travelling at an average velocity $\bar{v}$ (defined positive in the direction of the bearing), it will travel away from the 7th arc a distance $\Delta \bar{r}$ during time $\Delta T$ following

4 $$\begin{aligned} \Delta \bar{r} = \frac{c \Delta t}{1+c/\bar{v}}; \qquad \Delta T = \frac{\Delta t}{\bar{v}/c+1}. \end{aligned}$$

**Figure 14:**
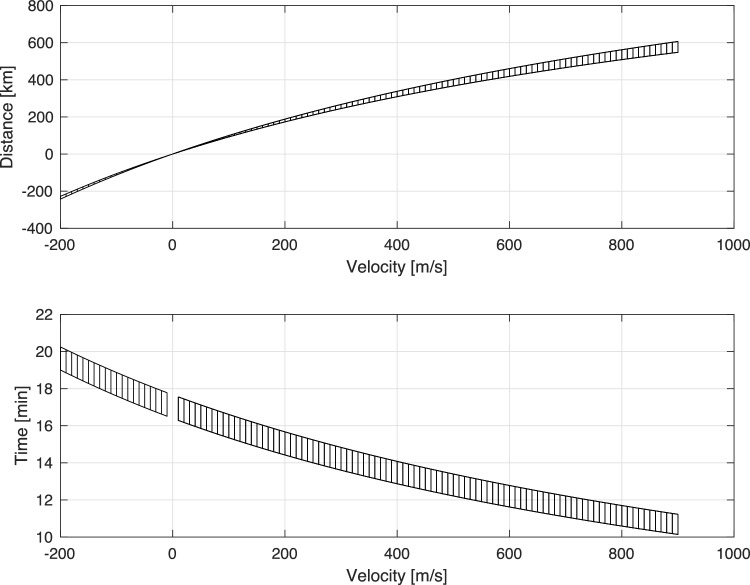
Distance (top) and time (bottom) that should have been travelled by MH370 after the last handshake (7th arc), as function of the average velocity (defined positive in the bearing direction.).

Figure 14 shows the possible combinations of distance and time that would have been travelled. Note that both $\Delta \bar{r}$ and $\bar{v}$ are vectors defined positive in the bearing direction, i.e., inwards the 7th arc. Illustration of the possible time duration and distance travelled (Eq. 4) as a function of the aircraft average velocity (defined positive in the direction of the bearing, travelling away). Hence, for this event to be associated with MH370, the aircraft must have remained in air for at least 10 min prior to impacting the water surface. Even for a considerably low average velocity of 1450 m/s, it would require the aircraft to remain in air about 13 min after the last handshake, and travel an extra distance of about 350 km. Finally, adding a tolerance of 0.5^∘^ in the bearing calculations provides the possible impact scenario depicted in Fig. 10. *Later impact* (blue area) is associated with lower average velocity travelled in opposite direction from H01W, and thus closer distance to the 7th arc, whereas *Earlier impact* is associated with spending a longer time in air with a higher average velocity resulting in travelling further away from the 7th arc, opposite to H01W. It is possible that the aircraft crash is outwards the 7th arc in the direction of H01W, but that will require the aircraft to last a much longer time in the air (above 17 min), before finally impacting the water.

It is worth noting that the signal with bearing $268.24^{\circ }$ recorded at 00:39:02 UTC is the only signal with $\Delta t = 0$, thus if originated at the exact 7th arc time, it would also be exact location. However, this signal is faint and would require further analysis, perhaps alongside the suggested field experiment.

**Table 1:** Time and bearing of signals recorded at H01W starting from 00:36 UTC.

Time (UTC)	Bearing	$\Delta t$ [min]	Time (UTC)	Bearing	$\Delta t$ [min]
00:38:29	261.24^∘^	$-3.68$	00:49:58	260.41^∘^	$+7.43$
**00:39:02**	**268.24**^∘^	$\textbf{0}$	00:51:50	320.40^∘^	$+12.72$
00:39:53	256.77^∘^	$-4.27$	00:52:36	329.51^∘^	$+11.10$
00:40:34	303.28^∘^	$+3.60$	00:52:36	324.26^∘^	$+12.65$
00:41:43	309.96^∘^	$+4.03$	00:52:50	274.84^∘^	$+14.15$
00:42:02	253.66^∘^	$+3.93$	00:53:31	257.58^∘^	$+9.75$
00:45:36	311.98^∘^	$+7.70$	00:53:38	315.46^∘^	$+15.32$
00:47:04	343.16^∘^	$-2.30$	**00:54:30**	**306.18**^∘^	$\mathbf{+17.10}$
00:48:55	291.13^∘^	$+11.77$	00:55:07	314.15^∘^	${ +16.95}$
00:49:41	234.67^∘^	–			

Two major signals where observed (bold), at 00:52 UTC with bearing $57^{\circ }$, and at 00:54:30 UTC with bearing $306.18^{\circ }$.

### Transmission losses due to changing bathymetry

**Figure 15:**
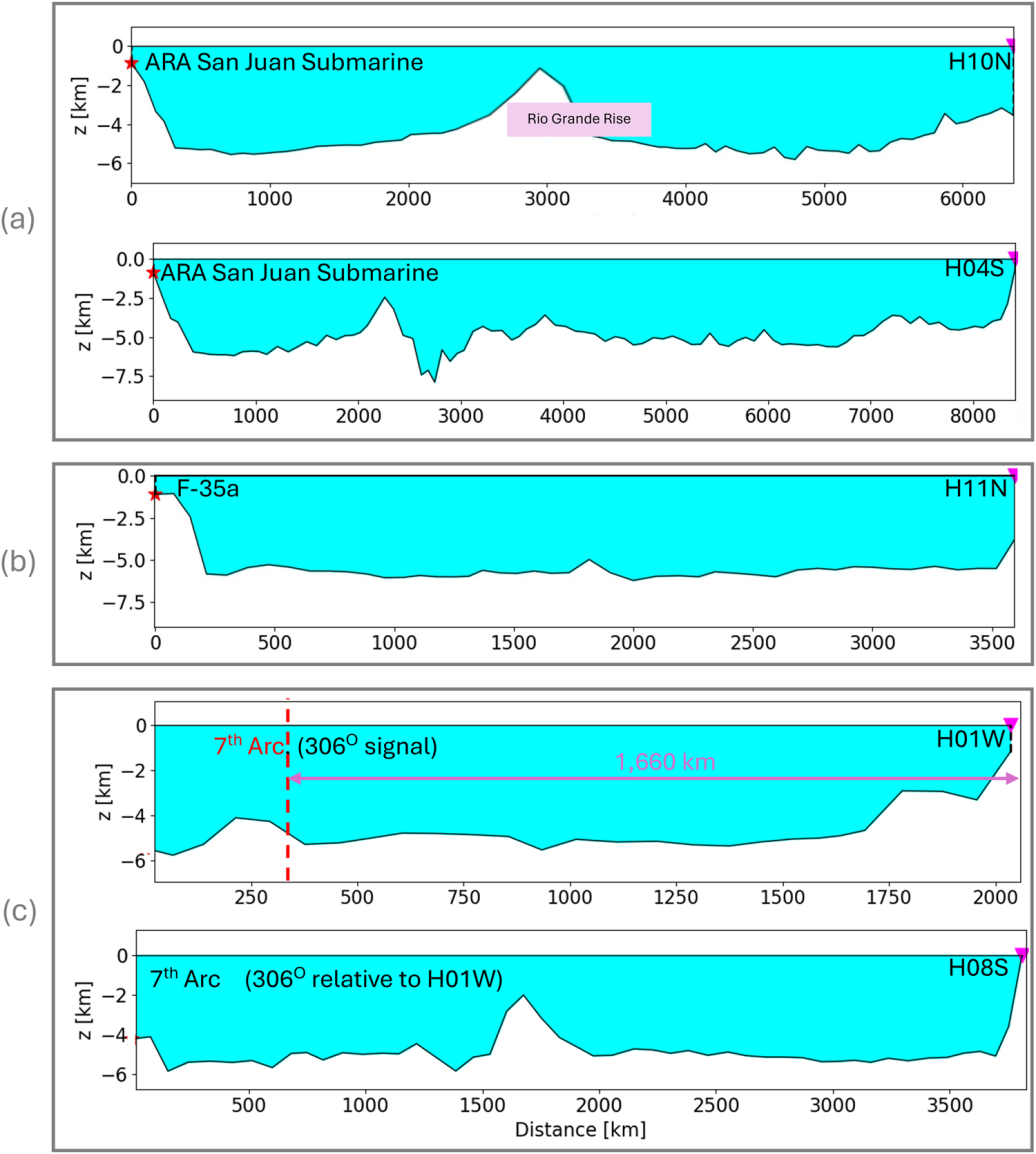
Transects of events to corresponding hydrophones. (**a**) ARA San Juan Submarine: a large barrier (Rio Grande Rise) half way to H10N; smaller barriers observed in the direction of H04S. (**b**) F-35a: clear path with no barriers observed. (**c**) Bearing 306^∘^: clear path with a minor barrier towards the end in the direction of H01W; a large barrier half way to H08S.

Ref.^4^ studied transmission losses in the case of the ARA San Juan Submarine. The authors reported a transmission loss reduction of 20 dB due to scattering by the Rio Grande Rise along H10N path. They asserted that the substantial bathymetric rise accounted for the higher recorded levels at H04S compared to H10N - noting that H04S is 8000 km far from the incident location, approximately 2000 km further than the path to H10N. The transects of these paths are shown in Fig. 15a.

Examining the transect of the F-35a case reveals a clear path, suggesting minimal losses due to bathymetric scattering and potentially explaining the distinct signal reception, see Fig. 15b. In the case of the 7th arc, the path to H01W is very similar to that of F-35a, though the distance is twice as short. Consequently, one would anticipate a clear distinct signal with minor losses due to a slight bathymetric rise near H01W, Fig. 15c. On the other hand, the path from the 7th arc to H08S presents a bathymetric barrier of comparable size, half way along the route, resembling the scenario of the ARA San Juan Submarine. Therefore, in the case of MH370, which arguably had a significantly less energetic compared to the Argentinian submarine, observing the signal at H08S becomes a challenging task, if feasible at all, due to transmission losses induced by bathymetry.

### Signals at the early stage of flight MH370

**Figure 16:**
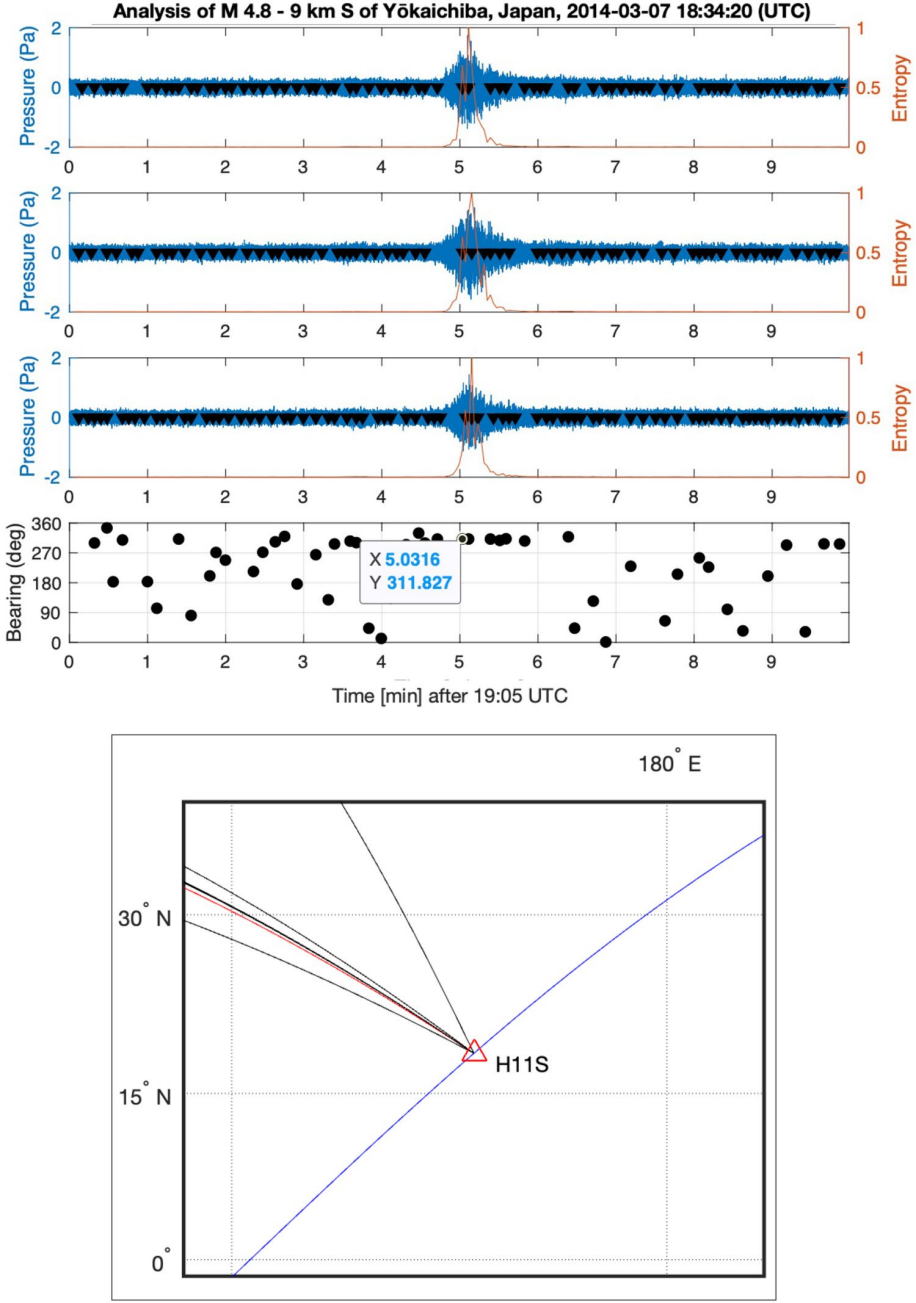
Bearing calculations of earthquake M 4.8–9 km S of Yōkaichiba, Japan, that erupted at 18:34:20 UTC on 7 March 2014 (Fig. 1). From top to bottom: recordings from three channels at station H11S between 19:05 and 19:15 UTC on March 7th 2014. Windowed entropy shows peaks (black triangles) where a transient signal is found. Each peak defines an event, for which bearing is calculated from differences in arrival times in the bearing subplot. The map demonstrates a perfect match with direction of the earthquake $312^{\circ }$.

**Figure 17:**
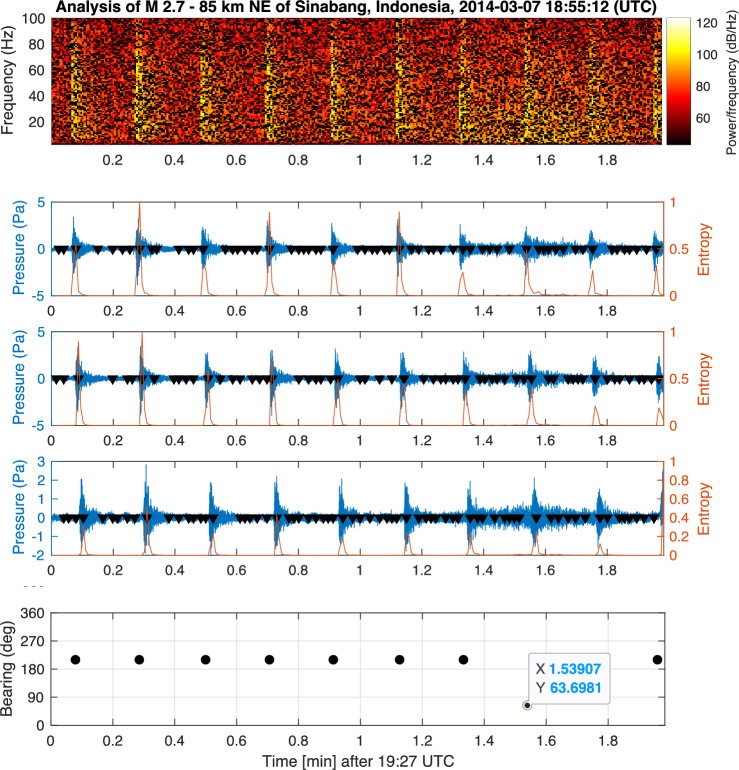
Bearing calculation of $210^{\circ }$ for the back noise, and of $63.7^{\circ }$ for earthquake M 2.7–85 km NE of Sinabang, Indonesia, that erupted at 18:55:12 UTC on 7 March 2014 (Fig. 2). From top to bottom: spectrogram and pressure recordings from three channels at station H08S between 19:27 and 19:29 UTC. Windowed entropy shows peaks (black triangles) where a transient signal is found. Each peak defines an event, for which bearing is calculated from differences in arrival times in the bearing subplot.

The main signal of interest at the early stage of the flight was recorded at H11S, with a bearing of $269^{\circ }$, as shown in Fig. 12. Signals that appear to be generated by airguns are also observed, at a bearing of $265^{\circ }$ relative to H11S. In addition, there are two earthquakes that erupted at time windows and locations related to the early stage of flight MH370. The first earthquake is a M 4.8–9 km South of Yōkaichiba, Japan, which erupted at 18:34:20 (UTC), on 7 March 2014. The earthquake epicentre is 3200 km away from H11S, which is a 36 min travelling distance for acoustic waves propagating at 1480 m/s. The bearing of the earthquake is $311.83^{\circ }$ as calculated from the geographic locations, which well matches the calculated bearing from the signal, ${ 311.827^{\circ }}$ (see Fig. 16), with almost no deviation. The second earthquake is the M 2.7–85 km NE of Sinabang, Indonesia, erupted at 18:55:12 (UTC), on 7 March 2014. The earthquake epicentre is 3000 km away from H08S, which is a 34 min travelling distance for acoustic waves propagating at 1480 m/s. The bearing of the earthquake is $67.12^{\circ }$ as calculated from the geographic locations. Due to the extremely high noise in the direction of $210^{\circ }$, and since most of the energy in earthquake seem to be between 2.5 and 6 Hz filtering allows calculating a bearing of $63.7^{\circ }$ with a relatively large deviation of $3.3^{\circ }$ (see Fig. 17), which is about 140 km off the epicentre.

### Supplementary Information

Supplementary Information.

## Data Availability

The hydroacoustic data analysed in this paper were obtained from the CTBTO and cannot be shared by the author to third parties, though can be requested directly from CTBTO. Data Availability Access to the IMS network’s data of the hydroacoustic stations is available to National Data Centres of the CTBTO and can be made available to others on request through the virtual Data Exploitation Center (vDEC) at https://www.ctbto.org/specials/vdec. Other data requests can be made by direct inquiries to the author.
