# Supplementary material for: Underwater acoustic analysis reveals unique pressure signals associated with aircraft crashes in the sea: revisiting MH370
Source: Sci Rep. 2024 May 2;14:10102. doi: 10.1038/s41598-024-60529-1 (PMC11065988; doi:10.1038/s41598-024-60529-1)
Supplement: Supplementary file 1 — Supplementary Information. [file 41598_2024_60529_MOESM1_ESM.pdf]

Underwater Acoustic Analysis Reveals Unique Pressure Signals Associated with Aircraft Crashes in the Sea: Revisiting MH370

Usama Kadri[1]

[1]School of Mathematics, Cardiff University, Cardiff, CF24 4AG, UK

*E-mail: kadriu@cardiff.ac.uk

Incidents Background

Malaysia Airlines Flight 370 (MH370)

On March 8, 2014, Malaysia Airlines Flight 370, a Boeing 777-200ER known as MH370, disappeared while flying from Kuala Lumpur, Malaysia to Beijing, China. MH370 had 227 passengers and 12 crew members on board. The disappearance of MH370 remains one of the most perplexing aviation mysteries in history. Shortly after takeoff, the aircraft's transponder and secondary radar contact were lost, making it difficult for air traffic control to track its location. Initial search efforts focused on the Gulf of Thailand and the South China Sea, but no debris or significant leads were found in those areas, not officially at least.

Later analysis of satellite data, known as Inmarsat, indicated that MH370 continued flying for several hours after its last confirmed position. The aircraft made a turn to the southwest, crossed the Malaysian Peninsula, and entered the southern Indian Ocean. Allegedly, MH370 went down near the last communication (or handshake) with the satellite, known as the 7th arc, at 00:19:29 UTC. The exact location where it went down remains unknown.

F-35A Stealth Jet Fighter

On April 9, 2019, a F-35A stealth jet fighter deployed in Japan crashed in the Pacific Ocean approximately 135 km east of Misawa Air Base in Aomori Prefecture, Japan, at 10:26:30 UTC (*1*). The coordinates of the crash site were approximately 40°30'10"N 142°04'37"E. The cause of the accident was attributed to spatial disorientation experienced by the pilot, which led to the loss of control of the aircraft. The aircraft impacted at the sea surface at about 300 m/s (1,080

km/h). The impact resulted in the generation of a hydroacoustic signal that was recorded at the Wake Island stations H11N and H11S, at a distance of about $3,340$ km (see figure 1).

Yemenia Flight 626

On June 30, 2009, Yemenia Flight 626, an Airbus A310, crashed into the Indian Ocean while flying from Sana'a, Yemen to Moroni, Comoros. The crash occurred around 01:50 local time (22:50 UTC on June 29, 2009) and the impact coordinates of the crash site were approximately 11°40'29.4"S, 43°16'39.6"E. The estimated impact speed of the aircraft was about 480 km/h, with the aircraft's nose striking first. The crash resulted in the loss of 152 of the 153 occupants, with only one survivor. Investigation revealed that the crash was likely due to pilot error, mechanical issues, and inadequate oversight by regulatory authorities (*2*).

Sriwijaya Air Flight 182

On January 9, 2021 a domestic passenger flight, Sriwijaya Air Flight 182, crashed into the Java Sea near the Thousand Islands, Indonesia (5°54'54" S, 107°07'06" E) at 07:40 UTC. The Boeing 737-500 (which is relatively smaller than MH370) lost control due to an auto-throttle malfunction, resulting in the loss of all 62 occupants (*3*). It is believed that the impact occurred at a high speed as the small scattered debris the wreckage indicated. Overall, the impact was sufficient to generate an acoustic signal that was recorded at Diego Garcia's station, H08S, at a distance of $4,694$ km (see figure 3).

Air France Flight 447

On June 1, 2009, Air France Flight 447, an Airbus A330, crashed into the Atlantic Ocean while flying from Rio de Janeiro, Brazil, to Paris, France. The crash occurred around 02:14 UTC and the impact coordinates of the crash site were approximately 3°03'57" N, 30°33'42" W. The estimated impact speed of the aircraft was about 282 km/h, with the aircraft's belly striking first. The crash resulted in the loss of all 228 aircraft occupants. Investigation into the accident revealed that a combination of technical problems due to malfunction of the airspeed sensors and incorrect inputs from the pilots contributed to the incident (*4*).

Transair Flight 810

On July 2, 2021, Transair Flight 810, a Boeing 737-200 cargo plane, crashed into the Pacific Ocean shortly after takeoff from Honulu lu, Hawaii, USA. The crash occurred around 11:46 UTC and the impact coordinates of the crash site were approximately 21°18'41" N, 157°54'16" W. The estimated impact speed of the aircraft was about 215 km/h. The two pilots on board were rescued by the US Coast Guard, but one of them later died due to injuries sustained during the crash. Investigation into the accident is ongoing (*5*).

Lion Air Flight 904

On April 13, 2013, Lion Air Flight 904, a Boeing 737-800, crashed into the sea while attempting to land at Bali’s Ngurah Rai International Airport. The impact coordinates of the crash site were approximately 8°45’00.96”S 115°09’01.01”E, and the impact time was around 07:10 UTC. The estimated impact speed of the aircraft was about 240 km/h. All 108 occupants survived the crash, but there were some injuries. Investigation revealed that the crash was caused by a combination of factors, including an incorrect approach procedure, pilot error, and problems with the aircraft’s autothrottle system (*6*).

AB Aviation Flight 1103

On February 26, 2022, AB Aviation Flight 1103, a Cessna 208D Grand Caravan, crashed into the sea about 2.5 kilometres from Mohéli Bandar Es Eslam Airport in the Comoros. The crash occurred at around 09:30 UTC (12°14’36.3552’S 43°46’50.6886”E. The crash resulted in the loss of all 14 occupants (*7*).

Air Asia Flight 8501

On December 28, 2014, Air Asia Flight 8501, an Airbus A320, crashed into the Java Sea while en route from Surabaya, Indonesia to Singapore. The crash occurred around 07:37 UTC and the impact coordinates of the crash site were approximately 05°50’36.0” S, 111°02’33.0” E. The estimated impact speed of the aircraft was about 274 km/h, with the aircraft’s nose dipping down and hitting the water first. The crash resulted in the loss of all 162 aircraft occupants. Investigation into the accident revealed that the pilots’ response to a malfunctioning rudder control system and their lack of understanding of the aircraft’s systems contributed to the incident (*8*).

Asiana Airlines Flight 991

On July 28, 2011, Asiana Airlines Flight 991, a Boeing 747-400F cargo aircraft, crashed into the sea off Jeju Island while en route from Seoul, South Korea, to Shanghai, China. The crash occurred around 04:11 Korean Standard time (19:14 UTC on July 27, 2011) and the impact coordinates of the crash site were approximately 33°15’04.56” N, 124°59’31.02” E. Just before impact the aircraft speed reached 429 knots (795 km/h), though the estimated impact speed of the aircraft was about 442 km/h. The crash resulted in the loss of the only two occupants on board, the pilot and co-pilot. Investigation into the accident revealed that a fire developed on or near the pallets containing dangerous goods causing some parts of the fuselage to separate from the aircraft in midair (*9*).

ARA San Juan Submarine

On November 15, 2017, The ARA San Juan, a TR-1700-class diesel-electric submarine, operated by the Argentine Navy, went missing during a exercise mission. Despite extensive search efforts, the submarine was found only a year later in the Atlantic Ocean at a depth of 907 metres (2,976 ft) at 45°56'59"S 59°46'22"W 20 km north northwest from a seismic anomaly reported by the CTBTO (*10*). The incident resulted in the loss of all 44 crew members.

MH370: The disappearance stage: Gulf of Thailand & South China Sea

MH370 departed Kuala Lumpur at 16:42 UTC, on 7 March 2014. Before reaching the Vietnamese border, waypoint IGARI, Kuala Lumpur air traffic control centre instructed the aircraft crew to contact the Vietnamese Control Centre, Ho Chi Minh. At 17:19 UTC the pilot-in-command of MH370 acknowledged the instructions by saying 'Good night Malaysia Three Seven Zero.', which was the last recorded radio transmission from MH370. After that time, any attempt to establishing communication with the crew had ended with failure.

At 17:22:57 UTC the aircraft was still en route, just a few seconds from performing the alleged sharp turn and go off route. The CTBTO hydroacoustic station H08 is at a distance of 3,800 km from the position of the aircraft, whereas H11 is as far as 6,900 km. Provided that an acoustic signal is travelling at an average speed between 1,450 m/s and 1,570 m/s, any signal from an impact should take between 40-43 minutes to reach H08 and 73-79 minutes to reach H11. Hence we are interested in signals that were recorded after 18:00 UTC, at H08 and 18:35 UTC at H11. However, note that since land separates the Gulf of Thailand and H08, the radiating signal could couple with the elastic seabed (as demonstrated in figure 7 top panel) travelling at a much faster speed of about 6,300 m/s during the 800 km inland journey, which would allow reaching H08S 7 minutes faster. Note that a wide spectrum of sound propagation speed has been considered to insure that include the entire spectrum of possible signals to ensures that no potential signal of interest is inadvertently overlooked (see Methods for more details).

The first observed signal is recorded at 17:54 UTC but only at H08S (figure S1 (a)). This signal is very weak, though its dispersion (illustrated in a white arrow), being similar to that associated with ARA San Juan, suggests an impulsive source much shorter than ARA San Juan signal recorded at H04S (figure 7 middle panel). Such an impulsive source generates signals of only a few seconds length, which makes measuring the bearing a challenging task (*11*).

Another signal is observed at 18:49 UTC, but again only at H08S (figure S1 (b)). The signal duration is at least twice as long compared to Lion Air Flight 904 (figure 5 (c), bottom panel), and about ten times longer than the signal from F-35a (figure 4 (a)).

The last observed signal of interest at H08S was recorded at 19:05 UTC (figure S1 (c)). Interestingly a corresponding signal has been also observed at H11S at 19:46 UTC (figure S1 (e)) with a bearing of 269° towards the Gulf of Thailand. The shape of the two signals indicates an

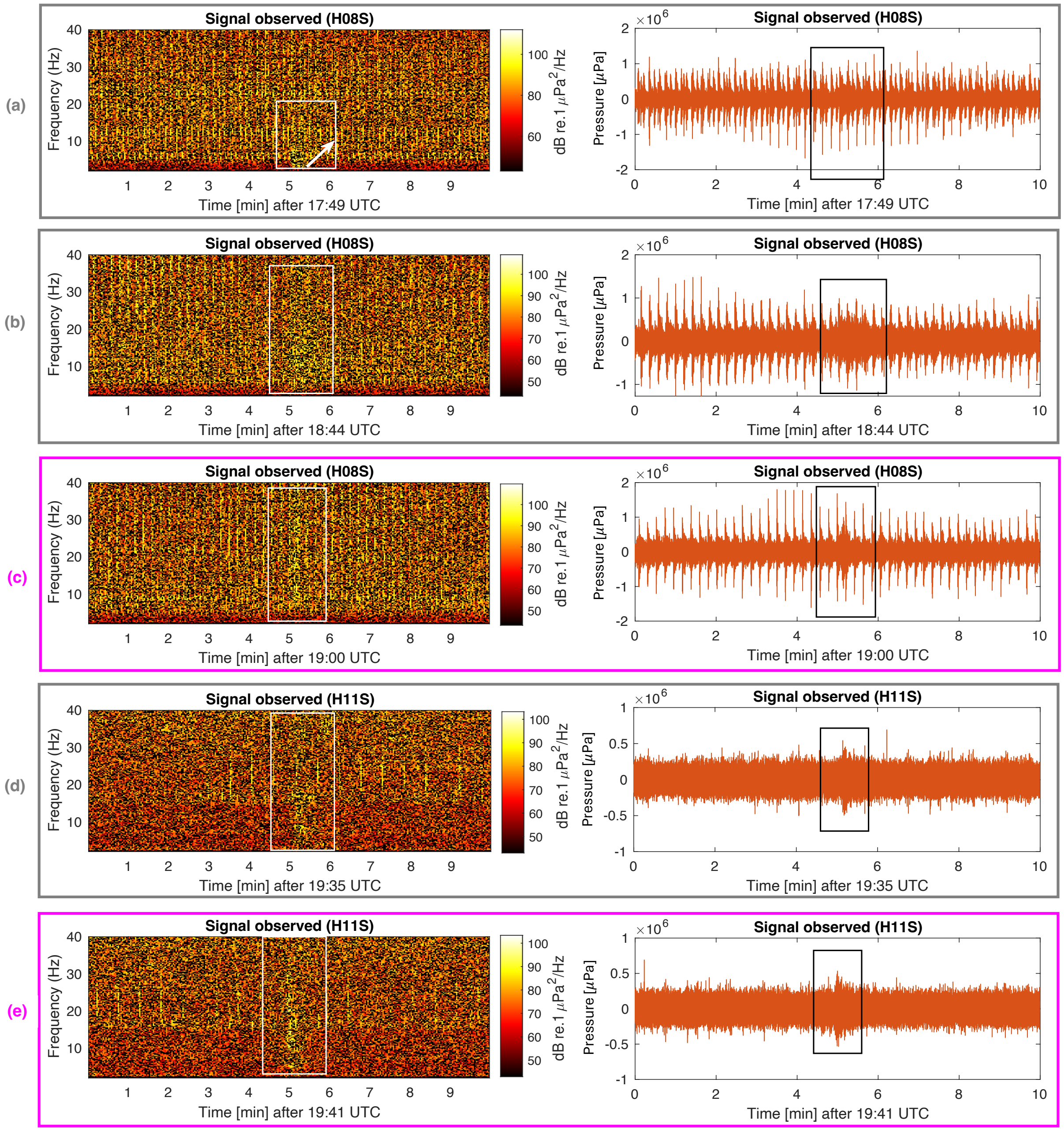

Figure S1: Spectrogram and pressure time series of signals observed at H08S and H11S on 7 March 2014 shortly after the disappearance of MH370. White arrow indicates dispersion; panels (c) and (e), highlighted in magenta, might be of the same source. Signal in panel (c) has a bearing of 269° relative to H11S, in the direction of the Gulf of Thailand.

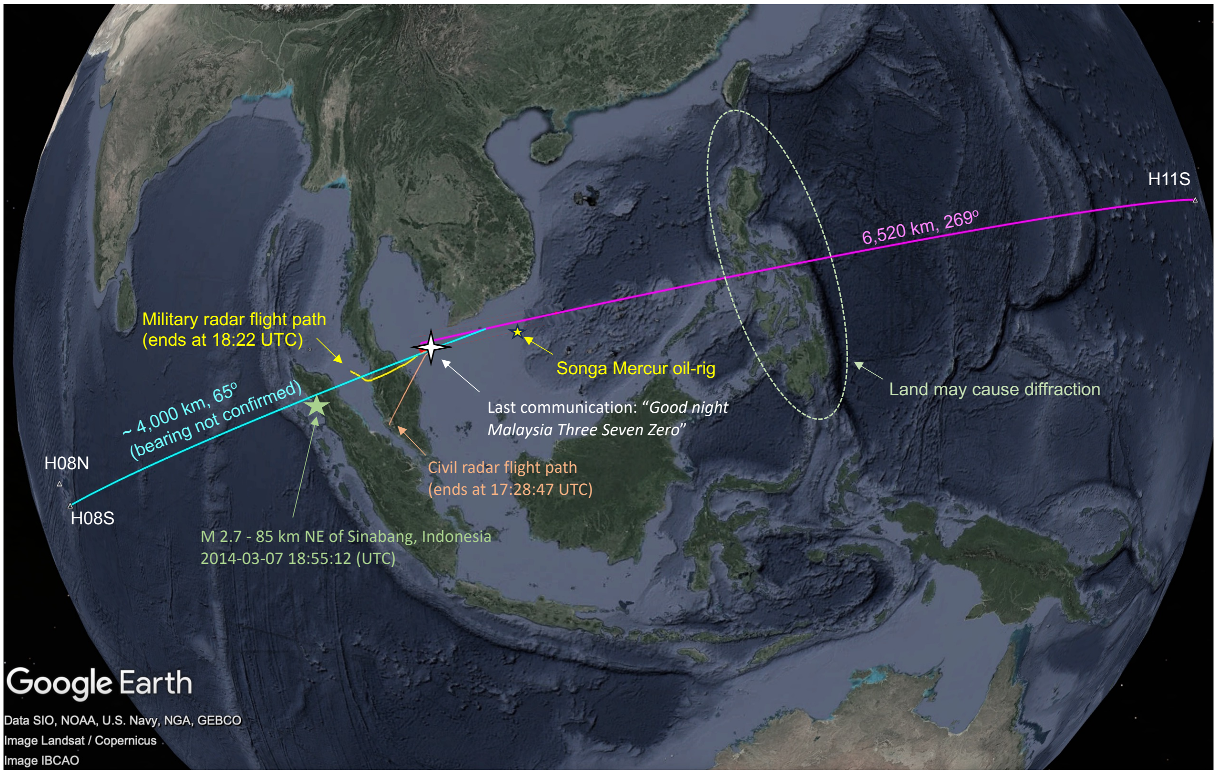

Figure S2: MH370 civil radar flight path (orange curve); location of MH370 after last communication with pilot-in-command (white star); military radar flight path (yellow curve); location of the CTBTO's hydroacoustic stations H08S, H08N and H11S (white triangles); distance and bearing of source relative to H11S (solid magenta); bearing and distance of source relative to H08S (solid cyan); possible diffraction by land (green dashed oval); M 2.7 earthquake NE) Sinabang, Indonesia (green star).

impulsive source, and crossing such long distances with transects that include large bathymetric irregularities would indicate a very high energy source. If the two signals on both stations are indeed related then the source is undoubtedly in the Gulf of Thailand, as shown in figure S2. However, it is notable that starting from 19:20 UTC repetitive signals (most likely to be airguns) are observed at H11S. Starting from 19:35 UTC the airgun signals become stronger. The signals from the airguns have a bearing of 265°, in the direction 250 km SSE away from the oil-rig Songa Mercur off Vung Tau, in the Gulf of Thailand. The question whether the signal of interest is caused by a related explosion or not is an open question, but anyhow timing suggests that it is unlikely to be associated with MH370. Note that, at 19:40 UTC there is another observed signal at H11S (figure S1 (d)), but it seems arriving from an opposite direction, 143°.

References

1. JASDF Air Staff Office. Factors of the F-35A fighter crash and recurrence prevention measures [in Japanese] Press Release. Ministry of Defense (2019a). https://www.mod.go.jp/asdf/news/houdou/ H31/20190610.pdf (Last accessed: 25 Jan 2022).

2. Final Report on the Accident on June 29, 2009 At sea off Moroni (Comoros) Of the Airbus A310-324 Registered 7O-ADJ Operated by the company Yemenia Airways (in French). National Civil Aviation and Meteorological Agency. 25 June 2013.

3. Final report on “Sriwijaya Air Flight 182”. Aviation Safety Network, Flight Safety Foundation, 2021. https://aviation-safety.net/database/record.php?id=20210109-0.

4. Final report On the accident on 1st June 2009 to the Airbus A330-203 registered F-GZCP operated by Air France flight AF 447 Rio de Janeiro – Paris (PDF), translated by BEA from French, Le Bourget: BEA Bureau of Enquiry and Analysis for Civil Aviation Safety, archived (PDF) from the original on 11 July 2012, retrieved 12 March 2017.

5. National Transportation Safety Board. (2022). Crash of Atlas Air Flight 8101 Boeing 747-400, N1217A, Near Anahuac, Texas, February 23, 2019. Retrieved from https://www.ntsb.gov/investigations/AccidentReports/Reports/AAR2201.pdf

6. Final Report No. KNKT.13.04.09.04, PT.Lion Mentari Airlines (Lion Air) Boeing 737 - 800;PK-LKS Ngurh Rai International Airport, Bali Republic of Indonesia 13 April 2013 (PDF). National Transportation Safety Committee. Archived from the original (PDF) on 29 November 2014. Retrieved 21 November 2014.

7. Ranter, Harro. “ASN Aircraft accident Cessna 208B Grand Caravan 5H-MZA Mohéli-Bandar es Eslam Airport (NWA)”. aviation-safety.net. Retrieved 10 March 2022.

8. National Transportation Safety Committee. (2015). Final Report: Aircraft Accident of AirAsia Flight QZ8501. Jakarta, Indonesia: Ministry of Transportation. CNN. (2014, December 28). AirAsia Flight QZ8501: What we know and don’t know. Retrieved from https://www.cnn.com/2014/12/28/world/asia/airasia-flight-qz8501-what-we-know/index.html

9. Crash Into The Sea After An In-Flight Fire, Asiana Airlines, Boeing 747-400F, HL7604 (PDF) (Report). Aviation and Railway Accident Investigation Board. 24 July 2015. Retrieved 11 May 2019.

10. Exclusivo: detalles del informe que recibió el Gobierno sobre la explosión en el submarino ARA San Juan”. InfoBAE. Retrieved 19 November 2018.

11. Dall'Osto, D.R. Source triangulation utilizing three-dimensional arrivals: application to the search for the ARA San Juan submarine. *Journal of the Acoustical Society of America*, **146**(1), 2104. https://doi.org/10.1121/1.5125251 (2019).
